# Metagenomics Reveals Microbial Diversity and Metabolic Potentials of Seawater and Surface Sediment From a Hadal Biosphere at the Yap Trench

**DOI:** 10.3389/fmicb.2018.02402

**Published:** 2018-10-12

**Authors:** Xinxu Zhang, Wei Xu, Yang Liu, Mingwei Cai, Zhuhua Luo, Meng Li

**Affiliations:** ^1^Institute for Advanced Study, Shenzhen University, Shenzhen, China; ^2^Key Laboratory of Marine Biogenetic Resources, Third Institute of Oceanography, State Oceanic Administration, Xiamen, China

**Keywords:** metagenomics, hadal biosphere, metabolic potential, microbial diversity, deep ocean

## Abstract

Hadal biosphere represents the deepest part of the ocean with water depth >6,000 m. Accumulating evidence suggests the existence of unique microbial communities dominated by heterotrophic processes in this environment. However, investigations of the microbial diversity and their metabolic potentials are limited because of technical constraints for sample collection. Here, we provide a detailed metagenomic analysis of three seawater samples at water depths 5,000–6,000 m below sea level (mbsl) and three surface sediment samples at water depths 4,435–6,578 mbsl at the Yap Trench of the western Pacific. Distinct microbial community compositions were observed with the dominance of Gammaproteobacteria in seawater and Thaumarchaeota in surface sediment. Comparative analysis of the genes involved in carbon, nitrogen and sulfur metabolisms revealed that heterotrophic processes (i.e., degradation of carbohydrates, hydrocarbons, and aromatics) are the most common microbial metabolisms in the seawater, while chemolithoautotrophic metabolisms such as ammonia oxidation with the HP/HB cycle for CO_2_ fixation probably dominated the surface sediment communities of the Yap Trench. Furthermore, abundant genes involved in stress response and metal resistance were both detected in the seawater and sediments, thus the enrichment of metal resistance genes is further hypothesized to be characteristic of the hadal microbial communities. Overall, this study sheds light on the metabolic versatility of microorganisms in the Yap Trench, their roles in carbon, nitrogen, and sulfur biogeochemical cycles, and how they have adapted to this unique hadal environment.

## Introduction

Hadal biosphere is known as the deepest part of the ocean, which is >6,000 m below sea level (mbsl). Due to technical constraints, little is known about the diversity and metabolic functions of microbial communities inhabiting this dark realm, which is among the least studied and most poorly explored of Earth's major biological habitats (Jamieson et al., 2010). It has been reported that the hadal trench is characterized by the extremely high hydrostatic pressure (>60 MPa) and isolated environments, while most of its physical and geochemical parameters such as temperature, salinity, and dissolved oxygen and organic carbon contents are similar with those from abyssal plains (4,000–6,000 mbsl) (Taira et al., 2005; Jamieson et al., 2010). In general, abundant heterotrophic microorganisms were detected in the hadal trenches, and heterotrophy is suggested to be enriched at greater depths of the trench axis due to the funneling effect resulting in an obvious accumulation of organic matters (Nunoura et al., 2015; Liu et al., 2017). Microbial transformation of bioavailable elements (e.g., carbon, nitrogen and sulfur) in hadal biosphere may have significant impact on global biogeochemical processes, and studies of these microorganisms would help to understand the microbial adaption strategies in this dark and high-pressure environment.

To date, most investigations focusing on hadal microbiology have taken place at the Mariana Trench located under the oligotrophic Pacific (6,000–10,257 mbsl), and diverse microbial communities have been detected and/or isolated by culture-dependent and -independent techniques spanning a large range of bacterial and archaea phyla (Yayanos et al., 1981; Nunoura et al., 2015). For example, some piezophilic strains of Gammaproteobacteria and Actinobacteria were isolated (Yayanos et al., 1981; Kato et al., 1998; Pathom-aree et al., 2006), and microbial communities dominated by Gammaproteobacteria, Bacteroidetes, Deferribacteres, and Thaumarchaeota were identified in the trench seawater (Nunoura et al., 2015; Tarn et al., 2016); higher rates of microbial carbon turnover and higher abundance of microbial cells occurred in sediments at the Challenger Deep of the Mariana Trench (10,900 mbsl) as compared to that at a nearby 6,000 mbsl site (9.7 × 10^6^ cells/cm^−3^ vs. 1.4 × 10^6^ cells/cm^−3^), revealing intensified microbial activity in this environment (Glud et al., 2013); analysis of 12 Parcubacteria single amplified genomes (SAGs) from the Challenger Deep sediments suggested their hadal adaptations to oxidative stress, polysaccharide modification and genes associated with respiratory nitrate reduction (León-Zayas et al., 2017). Another investigated hadal biosphere is the Puerto Rico Trench (PRT) in the northwestern Atlantic (6,000–8,300 mbsl) (George and Higgins, 1979). The microbial abundance of a 6,000 mbsl seawater sample in PRT was 1.1 × 10^4^ cells/mL, and the predominant microbial groups included Alphaproteobacteria, Betaproteobacteria, Gammaproteobacteria, and Planctomycetes (Eloe et al., 2011b); metagenomics further revealed the enrichment of genes including outer membrane porins, carboxylate transporters, sulfatases, and heavy metal resistance (Eloe et al., 2011a). Two identified SAGs clades of the Thaumarchaeota and SAR11 were found to possess new ecotype features of mixotrophic and piezophilic lifestyle, respectively (León-Zayas et al., 2015). In addition, the hadal seawater from the Japan Trench of the eutrophic Pacific (6,000–7,400 mbsl) showed distinct microbial community structures, which were dominated by Thaumarchaeota, Gammaproteobacteria, Alphaproteobacteria, and Bacteroidetes, and the seawater microbial abundance in this area was higher than that in the Mariana Trench (1.0 × 10^4^ cells/mL vs. 6.1 × 10^3^ cells/mL) (Nunoura et al., 2015, 2016). However, few high-throughput metagenomic surveys have been conducted to examine the functional potentials of the hadal microbiome (Eloe et al., 2011a; Carvalho, 2013).

The Yap Trench is located in the western Pacific with the water depth between 6,000 and 9,000 mbsl. The hydrographic feature of the Yap Trench is that the distance between the arc and trench axis (~50 km) is much less than that of other arc-trench systems, and a typical trench-trench junction is formed between north of the Yap Trench and the southern end of the Mariana Trench (Sato et al., 1997; Fujiwara et al., 2000; Ohara et al., 2002). Recent investigations of the Yap Trench have provided high-quality geomorphological, geochemical, and sedimentological data (Yang et al., 2017; Dong et al., 2018; Zhang et al., 2018), and a new starfish *Styracaster yapensis* was identified at ~6,500 mbsl (Zhang et al., 2017). However, limited information is available on the microbial diversity and their metabolic potentials in the hadal areas of the Yap Trench, thus the microbial processes such as heterotrophy and autotrophy in this environment are still unknown. In this study, we aim to reveal the microbial community structures and metabolic processes in the Yap Trench hadal biosphere, through comparative metagenomic analysis of three seawater samples (at depths between 5,000 and 6,000 mbsl) and three surface sediments (at depths between 4,435 and 6,578 mbsl) from the Yap Trench.

## Materials and methods

### Sample collection and geochemical analysis

All of the sediment and seawater samples were collected from the Yap Trench of the western Pacific during the 37th Dayang cruise in 2016 (Figure S1). Briefly, the sediment samples of Yap-D109-4435 and Yap-D113-6578 were collected by push core during *Jiaolong* submersible dives 109 and 113, respectively, and sample Yap-MC02-4568 was collected by multicore sampler. Only interior sections of the sediments were selected for microbiological study to avoid potential contamination as recommended elsewhere (Lever et al., 2013). The seawater samples of Yap-CTD02-5000, Yap-CTD02-6000, and Yap-CTD03-5700 were collected by CTD SBE911plus (Sea-Bird Electronics, USA), and 8 L of each seawater was filtered through a 0.22 μm-mesh membrane filter immediately after recovery onboard. All of the samples for microbiological analyses were stored at −80°C. Salinity and temperature were measured by a sensor (Sea & Sun, Germany) deployed on the CTD. The concentrations of ${\text{NH}}_{4}^{+}$, ${\text{NO}}_{2}^{-}$ and ${\text{PO}}_{4}^{3-}$ were measured by spectrophotometer, and the pH was measured by pH meter as described elsewhere (Zhang et al., 2016a). The dissolved O_2_ was analyzed based on the Winkler titration method (Winkler, 1888). Properties of these samples are detailed in Table 1.

**Table 1:** Description of the samples used in this study.

**Sample ID**	**Yap-D109-4435**	**Yap-MC02-4568**	**Yap-D113-6578**	**Yap-CTD02-5000**	**Yap-CTD03-5700**	**Yap-CTD02-6000**
Water depth (mbsl)	4,435	4,568	6,578	5,000	5,700	6,000
Sample discription	Surface sediment (0–2 cm), yellowish-brown clay with greenschist chips	Surface sediment (0–5 cm), yellowish-brown clay	Surface sediment (0–2 cm), yellowish-brown clay with basalt chips	Seawater	Seawater	Seawater
${\text{NH}}_{4}^{+}$ (μM)	NA	NA	0.69	0.90	0.13	0.88
${\text{NO}}_{2}^{-}$ (μM)	NA	NA	0	0	0.01	0.01
${\text{PO}}_{4}^{3-}$ (μM)	NA	NA	0.05	2.44	2.45	2.46
pH	NA	NA	7.8	7.9	7.9	7.9
Dissolved O_2_ (μM)	NA	NA	NA	173.6	176.4	182.5
Salinity	NA	NA	NA	34.7	34.7	34.3
Temperature (°C)	1.8	1.8	1.9	1.5	1.6	1.6
Clean data (Mbps)	56,883.2	37,032.5	56,993.6	58,321.3	48,241.9	65,196.7
Number of clean reads	379,221,052	246,883,510	379,957,476	386,233,704	319,482,552	431,766,380
Number of genomic bins	109	90	162	79	76	72
Number of contigs (>2 Kbps)	127,056	84,130	166,805	73,133	50,035	41,218
Number of ORFs	654,101	400,883	903,796	377,704	313,961	247,791
Bacteria (%, based on 16S rRNA genes from metagenome)	35.8	66.7	42.9	98.1	99.0	99.0
Archaea (%, based on 16S rRNA genes from metagenome)	63.6	32.7	56.3	1.8	1.0	1.0

*NA, not applicable*.

### DNA extraction and metagenomic sequencing

DNA was extracted using a MoBio PowerSoil® DNA Isolation Kit (MO BIO Laboratories, USA) according to the manufacturer's instructions with a few modifications. For sediments, ~0.5 g sample was added to a PowerBead Tube (MoBio PowerSoil® DNA Isolation Kit). For seawater, the 0.22 μm-mesh membrane was cut into ~0.2 cm^2^ pieces with a flame-sterilized scissors and added to a PowerBead Tube. Due to the challenging nature of sample retrieval and DNA recovery, whole genome amplification of the total DNA was performed with REPLI-g Single Cell Kits (Qiagen, Germany) following the manufacturer's protocol to obtain sufficient amounts of DNA for sequencing. Because the multiple displacement amplification (MDA) using phi29 DNA polymerase and random primers may introduce chimeric artifacts and lower genomic coverage (Kogawa et al., 2018), the MDA amplification was conducted in five separate reactions and the products were pooled for subsequent sequencing to reduce amplification biases. The amplified DNA was further purified using QIAamp DNA Mini Kit (Qiagen, Germany) according to the manufacturer's recommendations. Parallel blank controls, including sampling, DNA extraction and amplification controls, were performed with 0.22 μm-mesh membrane filtered Milli-Q water (18.2 MΩ; Millipore, USA). Sequencing was performed on Hiseq X Ten platform (Illumina, USA) using 2 × 150 bp pair-end technology.

### Metagenomic analysis

Clean reads were assembled into contigs using IDBA-UD (Version 1.1.1; Peng et al., 2012) with the parameters: -mink 65, -maxk 145, -steps 10. The ORFs within contigs were predicted by Prodigal with the “-p meta” option (Version 2.6.3; Hyatt et al., 2010). Taxonomic assignments of the Yap Trench metagenomes were performed using Metaxa2 (Version 2.1.3; Bengtsson-Palme et al., 2015) based on the identified 16S rRNA gene reads. Stress response genes were retrieved from the Genbank database (Benson et al., 2013) according to the collection of GeoChip 5.0 (Van Nostrand et al., 2016). Genes involved in various carbon, nitrogen, and sulfur metabolisms (i.e., hydrocarbons and aromatics degradation, carbon fixation cycle, nitrogen cycle, and sulfur cycle) were retrieved from FunGene (Version 9.5; Fish et al., 2013) and KEGG (Kanehisa et al., 2017). Genes involved in the HP/HB cycle were retrieved from Qin et al. (2017). The dbCAN web server (Version 6.0; Yin et al., 2012), and the BacMet database (Version 2.0; Pal et al., 2014) were used for the identification of carbohydrate-active genes and metal resistance genes, respectively. The amino acid sequences of the ORFs were subject to BLASTp searches (Version 2.5.0; Altschul et al., 1990) against the gene databases as described above, and matched genes with a maximum *e*-value of 10^−5^, minimum identity of 30%, and minimum query coverage of 50% were identified (Zhang et al., 2016b). The RPKM {reads per kilobase per million sequenced reads, RPKM = Mapped reads/[Gene length (Kb) × Total reads (million)]} was used to indicate the abundance of each gene, which was normalized to account for variations in gene length and dataset size as described elsewhere (Hu et al., 2013; Li et al., 2015, 2016; He et al., 2016; Liu Y. et al., 2018). Metagenome binning and taxonomic assignments were performed as described elsewhere (Liu Y. et al., 2018). Briefly, the initial binning was performed by setting different parameters of sensitivity and specificity in MetaBAT (Version 2.12.1; Kang et al., 2015). Then, all of the bins that retrieved from MetaBAT were pooled for post-dereplication by DAS Tool (Version 1.1; Sieber et al., 2018). The completeness and contamination of the genomes within bins were then estimated by CheckM (Version 1.0.7; Parks et al., 2015). Taxonomic assignments of the bins were performed using RefineM (Version 0.0.23; Parks et al., 2017) according to the percentage of gene taxonomy information in the reference database. Statistical significance between two samples was analyzed using Student's *t*-test by SPSS 22.0 software (IBM, USA), and differences were considered significant when *P* < 0.05. For the analysis of microbial community structures from some reported hadal environments, the 16S rRNA gene reads identified from a seawater metagenome of the PRT (6,000 mbsl) (Eloe et al., 2011a) and the deepest part of Mediterranean Sea (4,908 mbsl) (Smedile et al., 2013) were assigned using Metaxa2, respectively. The microbial community compositions of the six seawater from the Mariana Trench (5,000–10,257 mbsl) and the six seawater from the Japan Trench (4,989–7,407 mbsl) were obtained from Nunoura et al. (2015) and Nunoura et al. (2016), which were amplified with specific 16S rRNA gene primers and assigned using SILVA Ref NR database as described elsewhere.

### Phylogenetic analysis

Phylogenetic trees of the archaeal and bacterial *amoA* genes were constructed using IQ-TREE (Version 1.6.3; Nguyen et al., 2015) with ModelFinder (Kalyaanamoorthy et al., 2017), and the ultrafast bootstrapping was used to estimate the reliability of each branch with 1,000 resamples (Quang et al., 2013). Sequences covering the whole region of the *amoA* gene from type species and environmental clones from diverse environments (downloaded from GenBank) with sequence similarity to the Yap Trench sequences were included in the trees.

### Accession number

All sequence data (including raw reads and assembled contigs) have been deposited in the National Center for Biotechnology Information (NCBI) Sequence Read Archive under the accession number SRP151902.

## Results

### Microbial community composition

In general, a total number of reads between 246,883,510 (Yap-MC02-4568) and 431,766,380 (Yap-CTD02-6000) were retrieved from the six metagenomes in the Yap Trench after removing low-quality reads (Table 1). The reads were then assembled into 41,218–166,805 contigs with the contig length of >2 Kbps, and metagenomic binning resulted in an average of 98 genomes for each sample.

**Figure 1:**
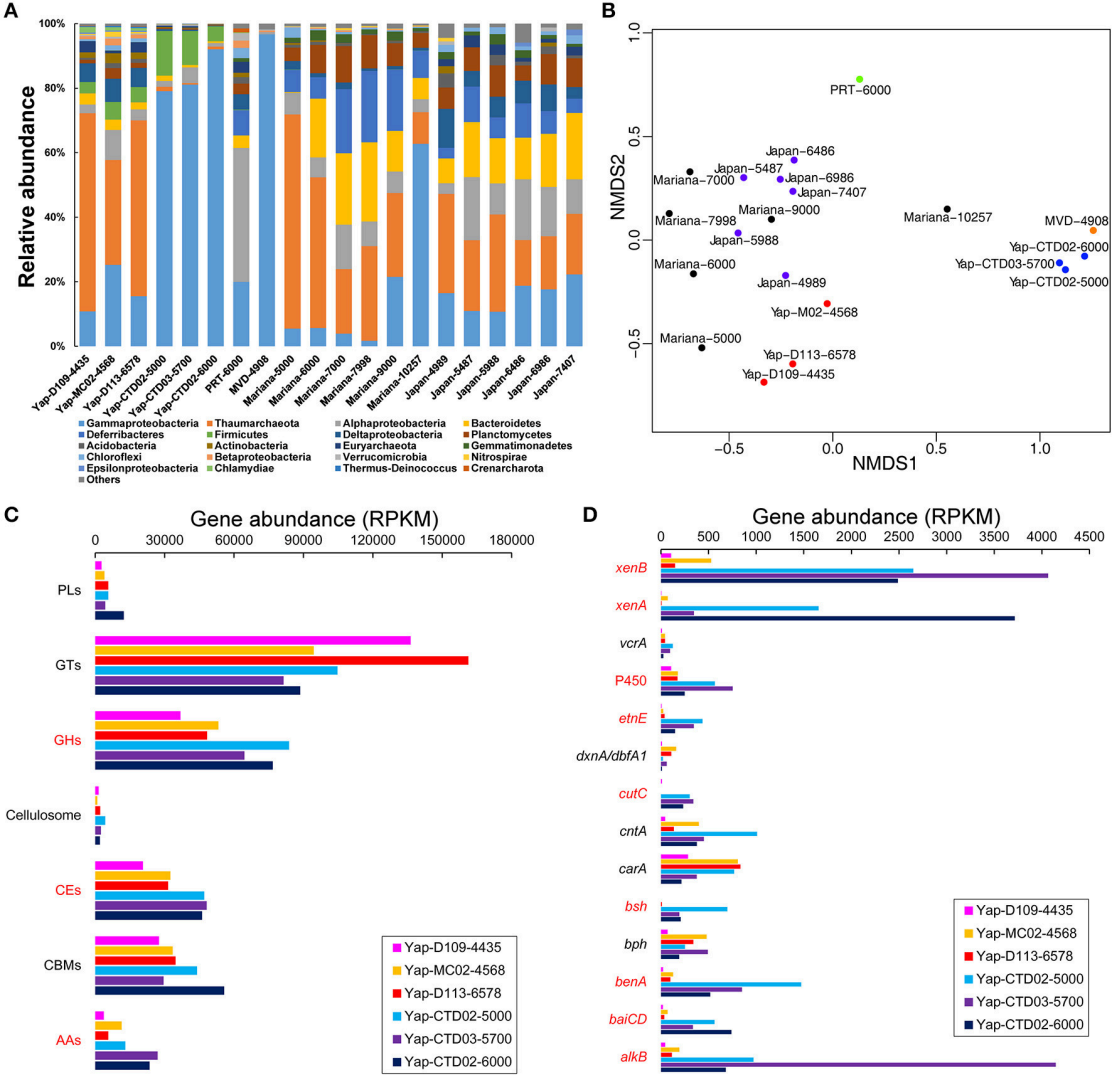
**(A)** Microbial community compositions of the Yap Trench metagenomes and some reported trench environments as determined by the 16S rRNA genes. Taxonomic assignments of the Yap Trench metagenomes were performed using Metaxa2 (Bengtsson-Palme et al., 2015) based on the identified 16S rRNA gene reads. **(B)** An NMDS plot showing variations in the microbial community structures of the samples from **(A)**. The distances were determined using the Bray-Curtis method with relative abundances of microorganisms at the phylum/class level. The red and blue filled circles are sediment and seawater samples of the Yap Trench, respectively. The green filled circle is seawater of the Puerto Rico Trench (PRT) (Eloe et al., 2011a). The orange filled circle is seawater of the Matapan-Vavilov Deep (MVD) (Smedile et al., 2013). The black filled circles are seawater of the Mariana Trench (Mariana) (Nunoura et al., 2015). The violet filled circles are seawater of the Japan Trench (Japan) (Nunoura et al., 2016). The numbers at the end of each sample ID indicate sampling depth (mbsl). Relative abundance of the microbial groups in each sample is listed in Table S1. **(C,D)** Gene abundances involved in the microbial degradation pathways of **(C)** carbohydrates and **(D)** hydrocarbons in the six metagenomes of the Yap Trench. AAs, auxiliary activities; CBMs, carbohydrate-binding modules; CEs, carbohydrate esterases; GHs, glycoside hydrolases; GTs, glycosyltransferases; PLs, polysaccharide lyases. *alkB*, alkane 1-monooxygenase; *baiCD*, 7α-hydroxy-3-oxo-Δ^4^-cholenoic acid oxidoreductase; *benA*, benzoate 1,2-dioxygenase hydroxylase; *bph*, biphenyl 2,3-dioxygenase; *bsh*, choloylglycine hydrolase; *carA*, carbazole 1,9a-dioxygenase; *cntA*, carnitine monooxygenase; *cutC*, choline trimethylamine-lyase; *dxnA/dbfA1*, dibenzofuran dioxygenase; *etnE*, 2-hydroxypropyl-CoM lyase; P450, cytochrome P450; *vcrA*, vinyl chloride reductase; *xenA*, xenobiotic reductase A; *xenB*, xenobiotic reductase B. The abbreviations in red indicate statistical significant between the three sediment and the three seawater metagenomes (*P* < 0.05). Detailed information of the gene abundances is listed in Table S2.

Based on the identified 16S rRNA gene reads from the six Yap Trench metagenomes, distinct microbial community structures were observed between the seawater and sediments, with the dominance of bacteria in the seawater (>98.1% in relative abundance) and archaea in the sediment (>32.7%), respectively (Table 1). For example, Gammaproteobacteria constituted up to 92.2% of the total microbial community in the hadal seawater of the Yap Trench, while Thaumarchaeota and Gammaproteobacteria were predominant groups in the surface sediments (48.4 and 16.9% in average relative abundance, respectively) (Figure 1A). Other major microbial groups in sediments included Deltaproteobacteria (6.2%), Alphaproteobacteria (5.0%), and Firmicutes (4.4%). In the seawater communities of the Yap Trench, the relative abundance of Gammaproteobacteria increased from 79.2 to 92.2% with water depth, while some other groups showed the opposite trend (i.e., Firmicutes and Bacteroidetes). The non-metric multidimensional scaling (NMDS) plot showed clear separation of microbial communities by the seawater and the sediments from the Yap Trench, and with the three samples in each group clustering together (Figure 1B). The microbial community compositions of the six Yap Trench samples were further investigated by retrieving the genomic bins from their metagenomes, and similar results were observed with those based on the identified 16S rRNA gene reads (Figure S2). The genomic bins that assigned to Gammaproteobacteria dominated the three seawater metagenomes, both in the relative abundance (number of reads mapped to a genomic bin; Figure S2a) and diversity (number of genomic bins in each taxon; Figure S2b). For the three sediments, bins that assigned to Thaumarchaeota were the predominant group with relative abundances between 40.8 and 49.1%, although their diversity was lower than other microbial groups in each sample.

### Carbon metabolism

#### Carbon degradation

Various genes involved in carbohydrate degradation were detected in all of the six Yap Trench metagenomes (e.g., glycoside hydrolases, carbohydrate esterases, and polysaccharide lyases), of which sediments and seawater samples showed distinct gene abundances (expressed as RPKM) toward different carbohydrates (Figure 1C). For example, the abundance of glycoside hydrolases (GHs, catalyzing the hydrolysis of O-, N-, and S-linked glycosides) in the sediments was lower than that in the seawater (average RPKM of 46,099 vs. 74,999; *P* < 0.05). The three seawater metagenomes of the Yap Trench also showed higher abundance of genes for carbohydrate esterases (CEs, catalyzing the de-O or de-N-acylation of substituted saccharides) and genes involved in auxiliary activities (AAs, helping glycoside hydrolases, polysaccharide lyases or carbohydrate esterases gain access to carbohydrates), respectively (*P* < 0.05). However, the abundance of the carbohydrate formation gene glycosyltransferases (GTs, catalyzing the formation of the glycosidic linkage to form a glycoside) appeared to be higher in the sediments than that in the seawater, although not significant (*P* > 0.05).

Furthermore, genes coding for the energy metabolisms involved in hydrocarbon degradation were identified, such as aliphatic hydrocarbons, aromatic hydrocarbons, and short-chain fatty acids (Figure 1D). Significantly higher abundance of genes for alkane and aromatic compound degradation (including *alkB, baiCD, benA, bsh, cutC, etnE*, and P450) often occurred in the seawater metagenomes than those in the sediments of the Yap Trench (Figure 1D; *P* < 0.05). Shallower depth of the seawater (Yap-CTD02-5000) appeared to possess more genes of *benA, bsh, carA, cntA, etnE*, and *vcrA* than the deeper ones (Yap-CTD03-5700 and Yap-CTD02-6000). The xenobiotic reductase genes (*xenA* and *xenB*) were also enriched in the seawater samples. Notably, the *alkB* gene involved in the first oxidation step in alkane degradation showed much higher abundance in sample Yap-CTD03-5700 than other samples.

In addition, diverse pathways of aromatic compound degradation were identified, in which genes coding enzymes for the degradation of benzoate, benzene, catechol ortho-cleavage, and xylene were more abundant in the seawater samples than those in the sediments (Figure S3; *P* < 0.05); however, the genes for phthalate degradation showed significantly higher abundance in the sediments than that in the seawater (*P* < 0.05). The naphthalene degradation genes were absent in all of the three seawater metagenomes, while they were detected in the sediments.

#### Carbon fixation

**Figure 2:**
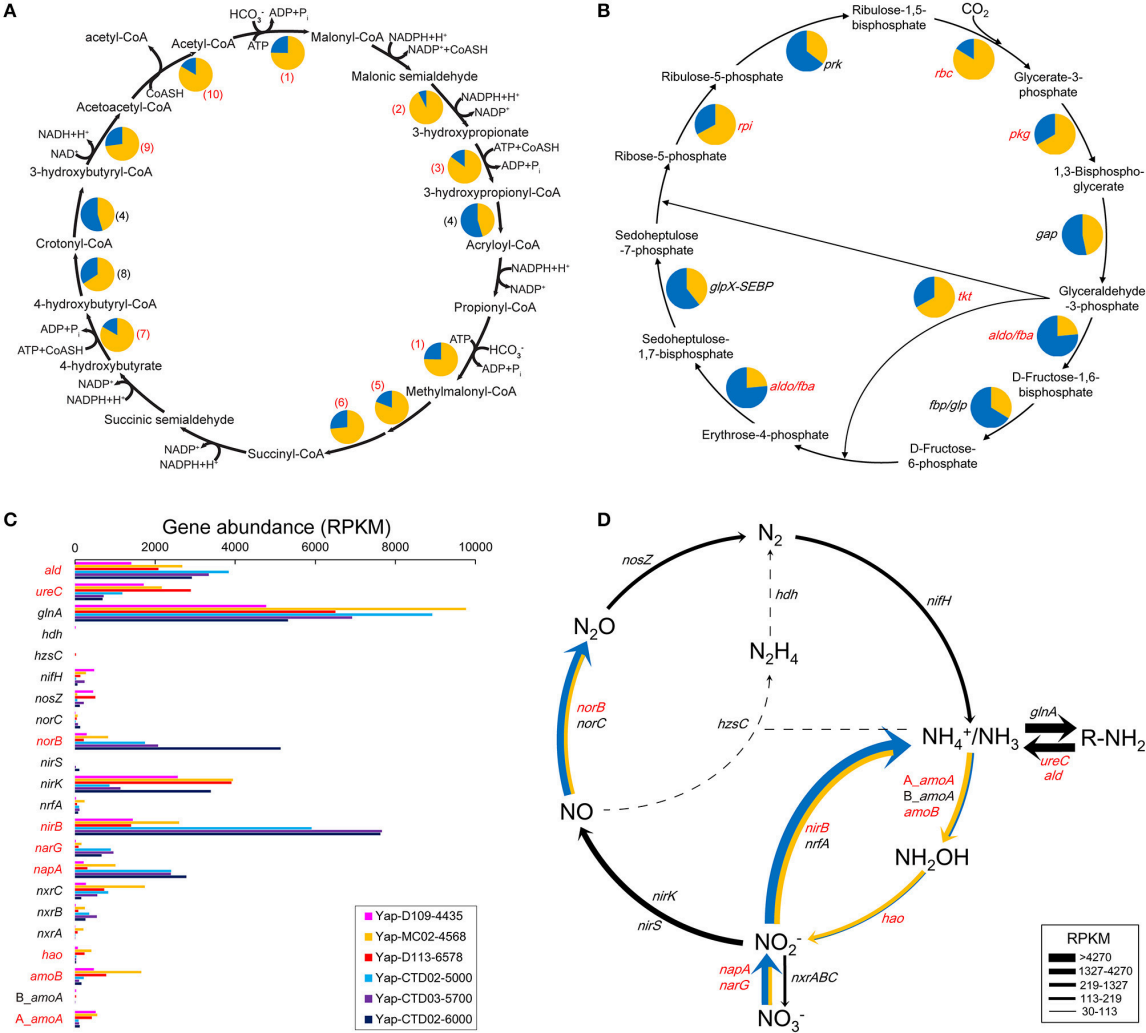
**(A,B)** Relative abundances of the genes involved in **(A)** the HP/HB cycle (modified from Qin et al., 2017) and **(B)** the Calvin cycle between the sediment and seawater metagenomes of the Yap Trench. The pie chart of each gene indicates its relative proportion between the three sediment (yellow) and the three seawater (blue) metagenomes. (1), acetyl-CoA-propionyl-CoA carboxylase; (2), malonyl-CoA reductase (NADPH); (3), 3-hydroxypropionyl-CoA synthetase (ADP-forming); (4), 3-hydroxypropionyl-CoA dehydratase; (5), methylmalonyl-CoA epimerase; (6), methylmalonyl-CoA mutase; (7), 4-hydroxybutyryl-CoA synthetase (ADP-forming); (8), 4-hydroxybutyryl-CoA dehydratase; (9), 3-hydroxybutyryl-CoA dehydrogenase; (10), propanoyl-CoA C-acyltransferase. *rbc*, ribulose-bisphosphate carboxylase; *pgk*, phosphoglycerate kinase; *gap*, glyceraldehyde-3-phosphate dehydrogenase; *aldo/fba*, fructose-bisphosphate aldolase; *fbp/glp*, fructose-1,6-bisphosphatase; *tkt*, transketolase; *glpX-SEBP*, sedoheptulose-bisphosphatase; *rpi*, ribose 5-phosphate isomerase; *prk*, phosphoribulokinase. The abundance of each gene is listed in Table S3. **(C)** Gene abundances involved in the microbial nitrogen cycle in the six metagenomes of the Yap Trench. *nif*, nitrogenase; *gln*, glutamine synthetase; *ald*, alanine dehydrogenase; *ure*, urease; A_*amo*, archaeal ammonia monooxygenase; B_*amo*, bacterial ammonia monooxygenase; *hao*, hydroxylamine oxidoreductase; *nxr*, nitrite oxidoreductase; *nap*, periplasmic nitrate reductase; *nar*, nitrate reductase; *nir*, nitrite reductase; *nor*, nitric oxide reductase; *nos*, nitrous oxide reductase; *hdh*, hydrazine dehydrogenase; *hzs*, hydrazine synthase; *nrf*, nitrite reductase. **(D)** The relative gene abundances between the sediment and seawater metagenomes as depicted by the width of solid lines. The yellow line indicates abundance higher in sediments, the blue line indicates higher in seawater, and the black line indicates no difference. The dashed line indicates little to no support for a pathway. The numbers/abbreviations in red indicate statistical significant between the three sediment and the three seawater metagenomes (*P* < 0.05).

Complete gene sets of two microbial carbon fixation pathways were detected in each of the sediment and seawater metagenome of the Yap Trench, including a variant of 3-hydroxypropionate/4-hydroxybutyrate cycle (HP/HB cycle) and the Calvin cycle (Figures 2A,B). Notably, all of the identified key genes of these two pathways showed much higher abundance in the sediments than that in the seawater (*P* < 0.05), including genes for acetyl-CoA-propionyl-CoA carboxylase, 3-hydroxypropionyl-CoA synthetase (ADP-forming), and 4-hydroxybutyryl-CoA synthetase (ADP-forming) in the HP/HB cycle (Figure 2A; Qin et al., 2017), and ribulose-bisphosphate carboxylase in the Calvin cycle (Figure 2B; Bar-Even et al., 2010), respectively. Based on the taxonomic information of the top BLASTp hit against the NCBI nr database, most of the key genes with the highest abundance in the HP/HB cycle were possibly derived from *Nitrosopumilus/Nitrosoarchaeum* of Thaumarchaeota, while those in the Calvin cycle were possibly from *Nitrosospira* of Betaproteobacteria.

### Nitrogen and sulfur metabolism

Key genes in the pathways of dissimilatory nitrate reduction to ammonia (DNRA), denitrification, nitrification, and nitrogen fixation were detected in both seawater and sediment metagenomes of the Yap Trench (Figure 2C). In general, the *nirB, napA/narG*, and *norB* genes encoding the enzymes for nitrate/nitrite removal (DNRA and denitrification) showed the highest abundance in the three seawater samples (*P* < 0.05), which were about four, five/nine, and seven folds higher than that in the sediment, respectively (Figure 2D). However, genes involved in nitrification (archaeal_*amoA*, bacterial_*amoA*, and *hao*) showed higher abundances in the sediment than those in the seawater (*P* < 0.05). In addition, abundant genes for ammonia assimilation (*glnA*) and ammonia production (*ureC* and *ald*) via organic matter transformations were also observed in all of the six metagenomes.

For sulfur metabolism, genes related to dissimilatory sulfate reduction and sulfur oxidation were detected in both seawater and sediment metagenomes (Table 2). All of the six metagenomes possessed the *asrABC* gene (encoding for anaerobic sulfite reductase, a key gene in dissimilatory sulfate reduction), but the *dsrAB* gene (which was reported more frequently from diverse environments) was only presented at low abundance in samples Yap-MC02-4568 and Yap-CTD03-5700. For sulfur oxidation, some genes involved in thiosulfate oxidation (*soxCD*), sulfide oxidation (*fccAB*), and sulfite oxidation (*soeC*) were detected in at least five samples. Other genes responsible for inorganic and/or organic sulfur transformations were also detected, including transformations of elemental sulfur/sulfide (*psrABC* and *sudA*) and thiosulfate/tetrathionate (*tsdA, ttrAB*, and *doxD*), and removal of sulfate from organic molecules, respectively (*FGly, atsK, sdsA1*, and *atsA*; Table 2).

**Table 2:** Gene abundances involved in the microbial sulfur cycle in the six metagenomes of the Yap Trench.

**Pathway**	**Gene**	**Gene description**	**Gene abundance (RPKM)**					
			**Yap-D109-4435**	**Yap-MC02-4568**	**Yap-D113-6578**	**Yap-CTD02-5000**	**Yap-CTD03-5700**	**Yap-CTD02-6000**
Dissimilatory sulfate reduction	*asrA*	Anaerobic sulfite reductase [iron-sulfur]	0.0	0.0	4.2	0.0	92.0	0.0
	*asrB*		1824.1	2183.4	4204.5	1073.2	1210.0	2775.1
	*asrC*		67.1	84.2	198.2	71.6	0.0	0.0
	*dsrAB*	Dissimilatory (bi)sulfite reductase [siroheme]	0.0	106.3	0.0	0.0	7.2	0.0
Assimilatory sulfate reduction	*cysH*	Phosphoadenosine phosphosulfate reductase	2285.2	2720.8	2976.2	2195.5	1584.8	3012.4
	*cysJ*	Sulfite reductase (NADPH) flavoprotein alpha-component	22.3	108.1	50.4	1674.9	1057.3	1386.1
	*cysNC*	Bifunctional enzyme CysN/CysC	5467.3	5079.2	6626.5	5198.5	3047.1	7206.2
	*sir*	Sulfite reductase [cytochrome]	4116.6	7740.4	3260.6	5028.9	5238.9	5489.2
	*fsr*	F420-dependent sulfite reductase [siroheme]	9.2	0.0	20.2	0.0	0.0	0.0
Sulfur oxidation	*soxC*	Sulfur-oxidizing multienzyme complex, subunits CD; Sulfanesulfur dehydrogenase [molybdenum/cytochrome]	157.0	1245.5	385.6	112.8	177.7	129.1
	*soxD*		179.7	1123.1	254.1	563.4	584.5	2855.1
	*fccA*	Flavocytochrome c/sulfide dehydrogenases [flavoprotein/cytochrome]	43.1	181.0	32.3	30.8	590.9	164.0
	*fccB*		581.8	492.6	492.3	776.0	1336.2	251.9
	*soeC*	Sulfite oxidizing enzyme [molybdenum]	7.4	64.3	6.8	0.0	21.7	4.8
Inorganic sulfur transformations	*psrA*	Polysulfide reductase [molybdenum]	213.6	1117.5	642.3	1087.9	2234.7	788.0
	*psrB*		0.0	134.2	0.0	104.0	110.2	80.4
	*psrC*		440.2	955.3	766.1	496.8	314.9	169.1
	*sudA*	Sulfhydrogenase/hydrogenase II (a.k.a. Sulfide dehydrogenase) [flavoprotein]	5512.8	8030.0	6505.1	7095.9	6203.9	10437.1
	*tsdA*	Thiosulfate dehydrogenase [diheme cytochrome]	16.6	0.0	13.8	30.3	59.8	160.3
	*ttrA*	Tetrathionate reductase [molybdenum]	10.6	68.6	95.6	291.6	193.6	116.8
	*ttrB*		557.2	2229.2	1297.7	3246.4	2588.4	1615.3
	*ttrC*		0.0	0.0	0.0	257.7	167.2	97.6
	*doxD*	Thiosulphate-quinone oxidoreductase	259.8	974.6	297.2	555.8	851.7	656.4
	*hydA*	Sulfhydrogenase/hydrogenase I [flavoprotein]	15.7	0.0	0.0	0.0	97.4	0.0
	*hydD*		0.0	0.0	0.0	0.0	113.1	0.0
	*phsA*	Thiosulfate reductase [molybdenum]	26.3	221.5	90.8	337.7	195.1	114.6
	*phsB*		0.0	11.5	17.1	436.1	316.4	195.2
	*phsC*		0.0	0.0	0.0	416.5	296.0	179.5
	*rdlA*	Rhodanese-like protein [rhodanase]	7.6	60.3	42.8	335.8	195.9	255.6
	*tetH*	Tetrathionate hydrolase	0.0	11.3	0.0	0.0	0.0	0.0
Organic sulfur transformations	*atsA*	Arylsulfohydrolase	360.3	2764.2	892.8	510.0	844.2	276.2
	*atsK*	Alkylsulfodioxygenase [dioxygenase]	4.7	55.2	52.4	194.0	97.3	100.5
	*cuyA*	Cysteate sulfo-lyase	200.0	954.1	380.7	825.1	579.4	258.4
	*FGly*	Formylglycine-dependent sulfhydrolase, sulfatase [formylglycine]	258.3	500.4	357.1	35.2	118.5	36.9
	*sdsA1*	Alkylsulfohydrolase	115.3	895.0	117.1	663.0	789.7	365.7
	*suyA*	Sulfolactate sulfo-lyase	7.7	87.7	23.5	137.6	63.2	60.9
	*suyB*		74.1	101.0	46.2	272.2	118.9	123.8
	*tauD*	Taurine dioxygenase	37.0	352.7	125.8	1103.7	620.7	377.4
	*xsc*	Sulfoacetaldehyde acetyltransferase	505.2	638.5	799.3	573.7	249.8	330.5

### Stress response and metal resistance genes

**Figure 3:**
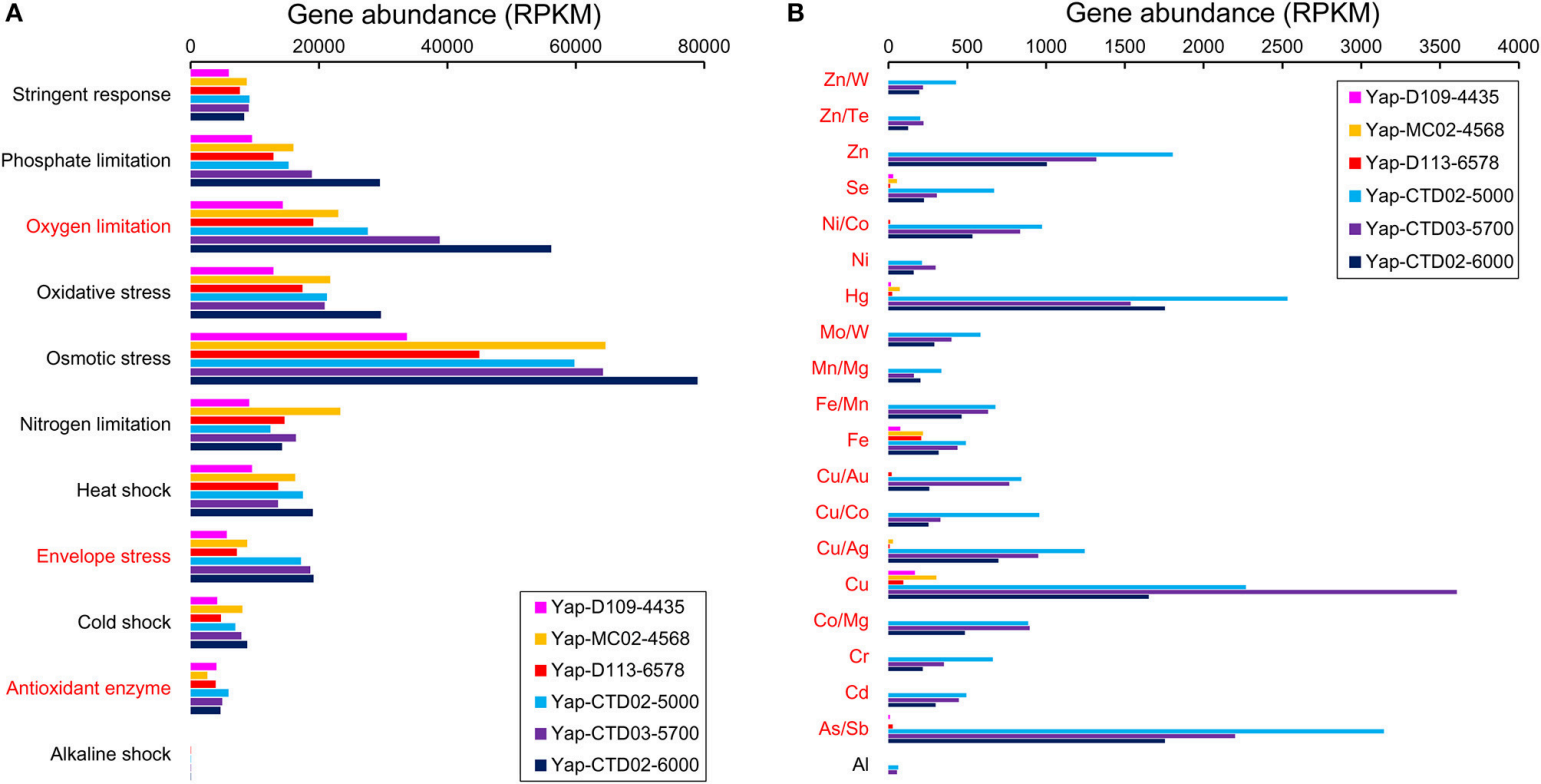
Gene abundances involved in **(A)** stress response and **(B)** metal resistance pathways in the six metagenomes of the Yap Trench. The metal elements are in abbreviated form. The genes that assigned to each stress response or metal resistance pathway are listed in Table S4. The pathways/metals in red indicate statistical significant between the three sediment and the three seawater metagenomes (*P* < 0.05).

In general, the Yap Trench metagenomes contained abundant stress-related genes that assigned to various stress response pathways, many of which did not show any difference between seawater and sediment (Figure 3A). Highest abundance of osmotic stress-related genes occurred among all of the stress-related pathways in each sample, followed by oxygen limitation and oxidative stress. Other genes for stress response detected in this study included phosphate and nitrogen limitations, envelope stress, stringent response, heat and cold shocks, and antioxidant enzyme. Interestingly, half of these stress response pathways in the seawater metagenomes showed an increased gene abundance along with sampling depth, including those associated with cold shock, envelope stress, osmotic stress, oxygen limitation, and phosphate limitation (Figure 3A).

Furthermore, seawater metagenomes of the Yap Trench possessed genes involved in resistance to some heavy metals (e.g., Hg, Cu, and Zn), but only few of them (Cu and Fe resistance) were detected with low abundances in the sediment (Figure 3B). For example, abundant metal resistance genes such as Hg/Co/Ni transporters (*merP, nrsD/nreB*; average RPKM = 540), Cu/Co/As/Sb efflux pumps (*golT, copA, corD, acr3*, and *arsB*; average RPKM = 369), and Cu/Zn/Co binding/oxidation proteins (*cutA* and *mco*; average RPKM = 449) were presented in the seawater metagenomes. Notably, the abundance of most of these metal resistance genes in the seawater metagenomes decreased along with the sampling depth, which was in contrast to the trend of some stress response genes as described above (Figure 3B).

## Discussion

### Microbial community structures in the yap trench

To reveal the features of hadal microbial community structures in the Yap Trench, we employed some reported microbiological data from different hadal environments, including a seawater metagenome from the PRT (6,000 mbsl) (Eloe et al., 2011a), a seawater metagenome from the deepest part of Mediterranean Sea (4,908 mbsl) (Smedile et al., 2013), six seawater samples from the Mariana Trench with 16S rRNA gene sequences (5,000–10,257 mbsl) (Nunoura et al., 2015), and six seawater from the Japan Trench with 16S rRNA gene sequences (4,989–7,407 mbsl) (Nunoura et al., 2016).

Seawater metagenomes from the Yap Trench possess similar microbial communities with those in the deepest parts of the Mediterranean Sea (MVD-4908) and the Mariana Trench (Mariana-10257) (Figure 1B), indicating that the Yap Trench seawater harbors some microbial communities that are characteristic of hadal environments. Notably, higher abundance of Gammaproteobacteria often occurred with increasing depth, as indicated in the seawater samples from the Yap Trench and the Mariana Trench (Figure 1A). Microorganisms from Gammaproteobacteria are cosmopolitan, which inhabit diverse deep-sea environments (e.g., hydrothermal vents Xie et al., 2011; Li et al., 2016, seafloor sediments Orcutt et al., 2011, and crustal biosphere Zhang et al., 2016b; Tully et al., 2017), and many of them are opportunistic heterotrophs that are also frequently detected in hadal environments (Liu et al., 2017). The retrieved genomic bins that showed the highest RPKM value in the Yap Trench seawater metagenomes were always assigned to *Pseudoalteromonas* or *Acinetobacter* of Gammaproteobacteria (Figure S2). *Pseudoalteromonas* spp. have been reported as a major constituent at the greatest depth of the Mariana Trench, the Kermadec Trench, and the New Britain Trench, in which many pure cultures in this genus are isolated (Tarn et al., 2016; Liu Q. et al., 2018; Peoples et al., 2018). This reveals that the *Pseudoalteromonas* spp. are viable in the hadal trenches during long geological periods. However, seawater microbial communities from 7,000 to 9,000 mbsl of the Mariana Trench and 5,487–7,407 mbsl of the Japan Trench were similar (Figure 1B), which were dominated by Thaumarchaeota (22.1% in average relative abundance), Bacteroidetes (17.5%), Gammaproteobacteria (13.5%), Alphaproteobacteria (12.8%), Deferribacteres (12.6%), and Planctomycetes (8.6%). The PRT seawater was characterized by its highest relative abundance of Alphaproteobacteria (40.5%) among all of these hadal environments.

For sediment metagenomes of the Yap Trench, they harbor similar microbial communities with the seawater samples from the shallow depths of the Mariana Trench (5,000 mbsl) and the Japan Trench (4,958 mbsl) (Figure 1B). The dominant microbial lineages in sediment of the Yap Trench did not show any correlation with water depth, indicating that factors other than depth (e.g., organic carbon contents or nutrients availability) influenced their community structures. The dominance of Thaumarchaeota in the Yap Trench sediments suggests the prevalence of chemolithoautotrophic ammonia oxidizers, which may play important roles in the nitrogen biogeochemical cycle (Walker et al., 2010; Dang et al., 2013).

In addition, Firmicutes appear to be unique to the Yap Trench environments, since few Firmicute sequences were detected in other hadal samples based on the 16S rRNA genes (Figure 1A). This is further confirmed by the retrieval of some *Exiguobacterium* (designated to Firmicutes) genomic bins in the three seawater metagenomes of the Yap Trench (Figure S2), suggesting that they may survive and adapt to various extreme environments including hadal trenches with high hydrostatic pressure (Vishnivetskaya et al., 2009).

### Heterotrophy vs. autotrophy

Heterotrophic metabolism is assumed as one of the most common microbial metabolisms in the Yap Trench seawater and sediment, because their microbial communities were dominated by potential heterotrophic microorganisms such as Gammaproteobacteria (Figure 1A, Figure S2), and various genes involved in the microbial degradation of carbohydrates, hydrocarbons, and aromatic compounds were detected (Figures 1C,D, Figure S3). This was further confirmed by the observation of enhanced process of organic matter deposition and higher microbial cell abundance at the Mariana Trench as compared to its neighboring abyssal counterparts (Glud et al., 2013). Due to the typical “V-shape” topography of the trenches, it may create a funneling effect resulting in an obvious accumulation of organic matters, which are originated from sinking particulates from the upper ocean, terrestrial inputs, chemosynthesis from the dark ocean, or even cell lysates at the trench axis (Jover et al., 2014). These materials are then migrating slowly toward the deepest trench axis driven by gravity (Ichino et al., 2015; Jamieson, 2015; Liu et al., 2017). In these heterotrophic processes, oxygen might be a primary electron acceptor as oxygen was not limited in the Yap Trench seawater and surface sediments (Table 1), although some micro-niches in the sediment could be anaerobic (Jørgensen, 1977). In addition, nitrate/nitrite or sulfate could be alternative electron acceptors coupling the oxidation of organic matters, since complete gene sets in the pathways of denitrification (*nirK, norBC*, and *nosZ*), DNRA (*nirB* and *nrfA*), and some key gens in dissimilatory sulfate reduction (*asrABC* and/or *dsrAB*) were detected in the Yap Trench metagenomes (Figure 2C, Table 2).

**Figure 4:**
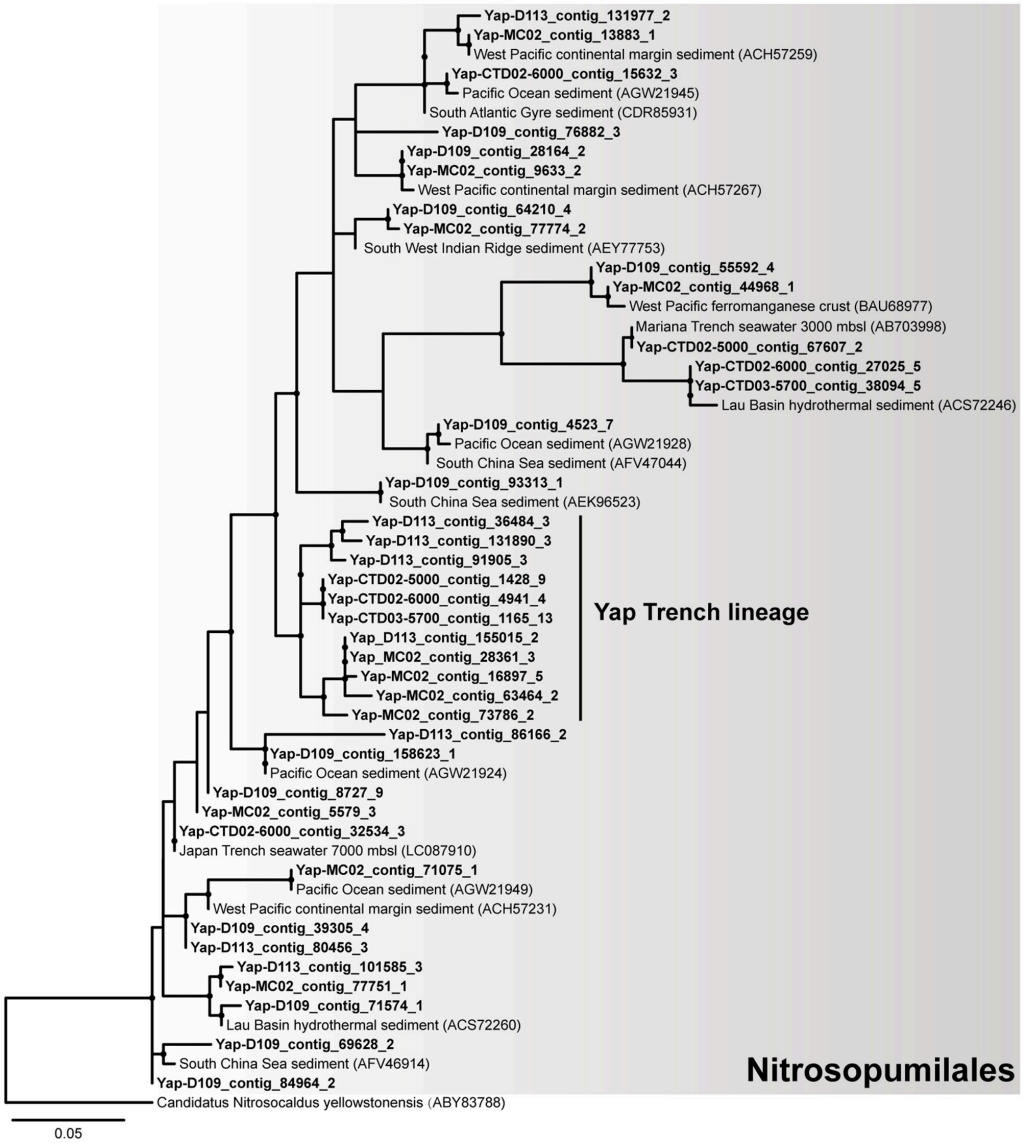
The phylogenetic tree of archaeal *amoA* genes from the six metagenomes of the Yap Trench. The sequences retrieved from this study are highlighted in bold font. Only those with >60 bootstrap values are shown as filled circles at each branch. The *amoA* gene of Candidatus Nitrosocaldus yellowstonensis (ABY83788) is used as the outgroup. The scale bar indicates 0.05 amino acid substitutions per site.

**Figure 5:**
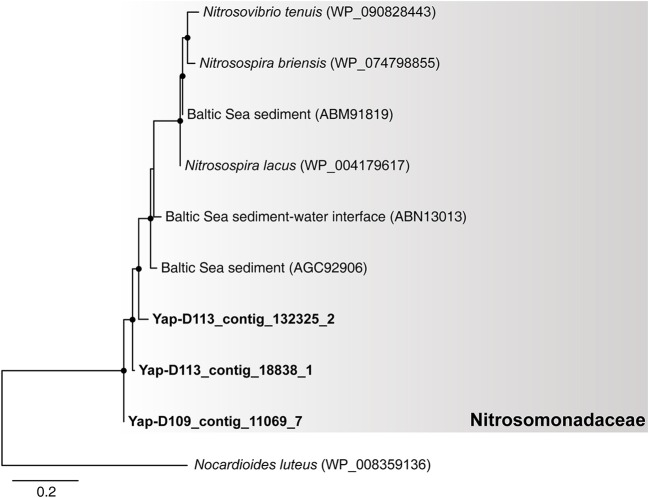
The phylogenetic tree of bacterial *amoA* genes from the six metagenomes of the Yap Trench. The sequences retrieved from this study are highlighted in bold font. Only those with >60 bootstrap values are shown as filled circles at each branch. The *pmoA* gene of *Nocardioides luteus* (WP_008359136) is used as the outgroup. The scale bar indicates 0.2 amino acid substitutions per site.

Notably, our results suggest that autotrophic processes such as ammonia oxidation probably dominate the surface sediment communities of the Yap Trench, which could be supported by the following few lines of evidence. First, Thaumarchaeota and Betaproteobacteria were significantly enriched for their 16S rRNA genes, genomic bins, and carbon fixation genes in the sediment as compared to those in the seawater (Figure 1A, Figures S2, S4, Table S5; *P* < 0.05). Second, phylogenetic analysis of the *amoA* genes in the sediment showed that all archaeal *amoA* genes fall within the Nitrosopumilales lineage of Thaumarchaeota (as proposed by Alves et al., 2018) (Figure 4), and all bacterial *amoA* genes were closely related to *Nitrosospira* of Nitrosomonadaceae (Figure 5). Third, most of the key genes with highest abundance in the HP/HB cycle and the Calvin cycle were also possibly derived from *Nitrosopumilus/Nitrosoarchaeum* of Thaumarchaeota and *Nitrosospira* of Betaproteobacteria, respectively. In total, because it is well-known that most members from Nitrosopumilales and *Nitrosospira* are autotrophic ammonia oxidizers (Norton et al., 2008; Könneke et al., 2014), autotrophic processes such as ammonia oxidation are probably supported by the availability of ammonia in the sediments of the Yap Trench. As for the sources of ammonia, it could be provided through two ways as inferred by our metagenomic data, including (1) decomposition of nitrogenous organic matters (Nunoura et al., 2015), and (2) dissimilatory nitrate reduction to ammonia (DNRA) (Figure 2D). However, we speculate that the Yap Trench sediment was a nutrient-limited environment, and the *in situ* concentration and availability of ammonia and organic matters were characterized by constantly low supply (Martens-Habbena et al., 2009; Sintes et al., 2013). As a result, autotrophic Thaumarchaeota prevail in the sediments by using the HP/HB cycle for CO_2_ fixation, which is reported as the most energy-efficient aerobic autotrophic pathway yet characterized (Könneke et al., 2014). This might also be explained by the “V-shape” or funneling effect of the trenches. During the process of sediment migration from the upper ocean to the deepest trench axis, liable organic matters and nutrients are consumed rapidly by microorganisms, while materials such as refractory organics (lignin, humic acids, or aromatic compounds), lithogenic minerals (carbonate or silicate rocks), and heavy metals (Cu or Hg) were preserved, which creates an oligotrophic environment at the bottom of the trench eventually. Unfortunately, we were not able to reveal the controlling factors that determine the dominance of autotrophic Thaumarchaeota in the Yap Trench sediment due to the limit of this study.

### Stress response and metal resistance

The observation of some stress response genes in the Yap Trench (such as osmotic stress, oxidative stress, oxygen limitation, and phosphate limitation) suggests that high pressure and/or nutrient availability could be a stressor for living microorganisms in the hadal biosphere, and these microorganisms could adapt to this environment by increasing the abundance of corresponding stress response genes (Figure 3A). For example, abundant osmotic stress genes could adjust the microbial cellular concentration of osmolytes or compatible solutes (Kempf and Bremer, 1998), and oxidative stress and oxygen limitation genes improve the imbalance between the production and scavenging of reactive oxygen species and respond to low oxygen level (Mostertz et al., 2004). The abundance of phosphate and nitrogen limitation genes in the Yap Trench further supports the hypothesis that phosphorus and/or nitrogen are common limiting nutrients in marine environments (Moore et al., 2013; Ward, 2013; Zhang et al., 2016a). In addition, water depth might be an important factor that controls the abundance of some major stress response pathways in the Yap Trench (including osmotic stress and oxygen limitation), indicating intensified stresses for microorganisms toward deeper regions of the Yap Trench.

The detection of various metal resistance genes (e.g., Hg, Cu, Fe/Co/Ni, and As/Sb resistance) in the Yap Trench metagenomes is intriguing (Figure 3B), since these genes are mainly represented in contaminated environments (Hemme et al., 2010) or waste water treatment systems (Di Cesare et al., 2016). However, this finding could be inferred from three previous reports that (1) abundant heavy metal resistance genes were detected in a 6,000 mbsl seawater metagenome of the PRT (Eloe et al., 2011a), (2) metal resistance genes such as Co/Zn/Cd efflux system components were enriched in a seawater metagenome of the deepest part of the Mediterranean Sea (Matapan-Vavilov Deep, 4,908 mbsl), and (3) an enrichment of heavy metal resistance genes occurred in the genome of the deep *Alteromonas macleodii* ecotype as compared to the shallow-water counterparts (Ivars-Martinez et al., 2008). As discussed in the preceding section, the enrichment of various metal resistance genes could also be explained by the funneling effect of the trenches, which have accumulated large amount of heavy metals in the hadal trenches. This is further confirmed by the observation of increased concentrations of Cu, Fe/Ni, and/or Zn in the deep-sea water as compared to those in the surface seawater from the Atlantic Ocean and the Pacific Ocean, respectively (Coale and Bruland, 1988; Moffett and Dupont, 2007; Little et al., 2015). Combined with our findings and previous reports, we hypothesize that the enrichment of metal resistance genes is characteristic of the hadal microbial communities.

## Conclusion

This study reveals that the hadal seawater and surface sediment of the Yap Trench harbored distinct microbial populations and metabolic processes, which were dominated by heterotrophic Gammaproteobacteria and autotrophic Thaumarchaeota, respectively. In the seawater, abundant genes involved in the degradation of various types of carbohydrates, hydrocarbons, and aromatics were detected, showing their potentials to use these organic carbon sources. In the surface sediments, chemolithoautotrophic ammonia oxidation with the HP/HB cycle for CO_2_ fixation is possibly a dominant process in the Yap Trench. Moreover, the detection/enrichment of genes involved in stress response and metal resistance in the seawater and sediment of the Yap Trench suggests special adaptation strategies of the hadal microorganisms toward high pressure and/or nutrient availability, and the enrichment of metal resistance genes is a hypothesized characteristics of the hadal seawater microbial communities. This study confirms the metabolic versatility of a unique hadal microbial biosphere hosted in the deepest part of the ocean, filling a knowledge gap about the extent of life on Earth. Although autotrophic metabolisms are indicated in the sediment of the Yap Trench in this study, it is still an open question due to the limited data. Future studies including *in situ* microbiological and geochemical measurements are needed to determine the microbial metabolic activities and specific contributions of autotrophy vs. heterotrophy in the hadal biosphere.

## Author contributions

XZ, ML, ZL, and WX designed and performed the experiments, analyzed the data and wrote the manuscript. YL and MC analyzed the data. All authors commented on the manuscript.

### Conflict of interest statement

The authors declare that the research was conducted in the absence of any commercial or financial relationships that could be construed as a potential conflict of interest.
