# Supplementary material for: Metagenomics Reveals Microbial Diversity and Metabolic Potentials of Seawater and Surface Sediment From a Hadal Biosphere at the Yap Trench
Source: Front Microbiol. 2018 Oct 12;9:2402. doi: 10.3389/fmicb.2018.02402 (PMC6194347; doi:10.3389/fmicb.2018.02402)
Supplement: Supplementary file 1 [file Data_Sheet_1.pdf]

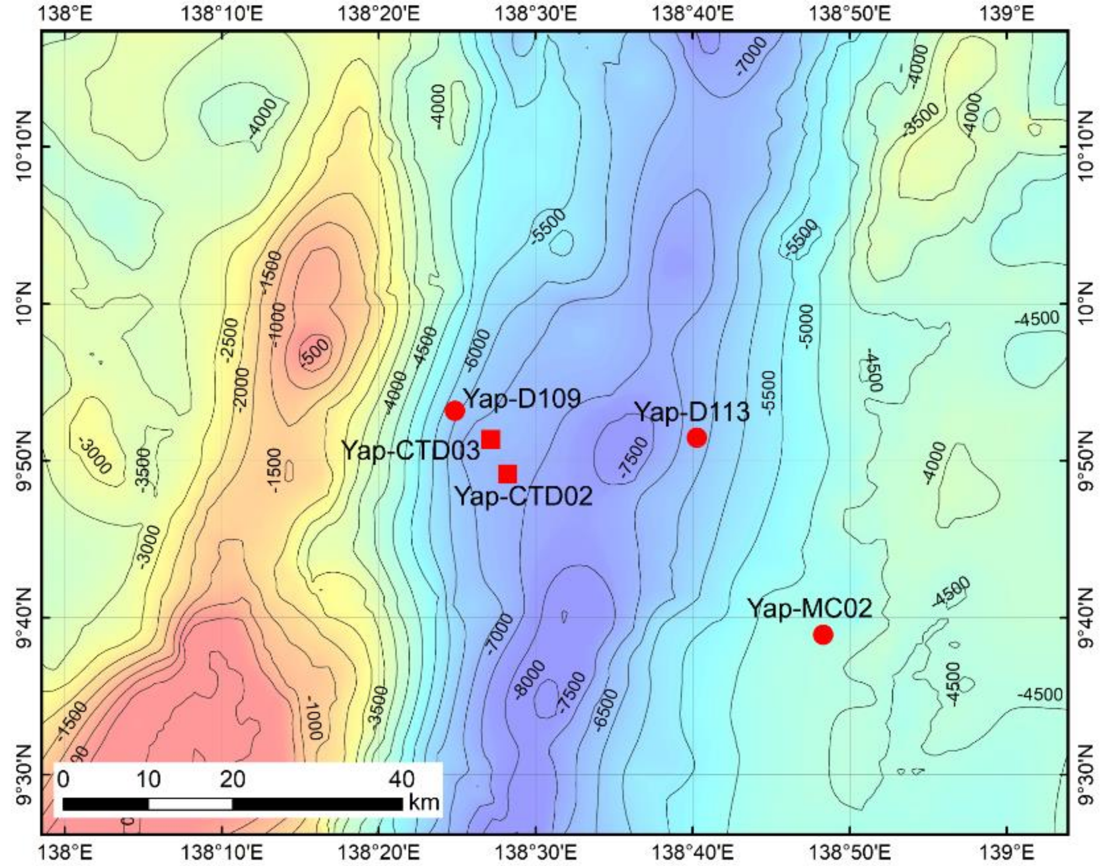

Figure S1. Sampling location of the Yap Trench. The red solid circles are sediment collection sites, and the red squares are seawater collection sites.

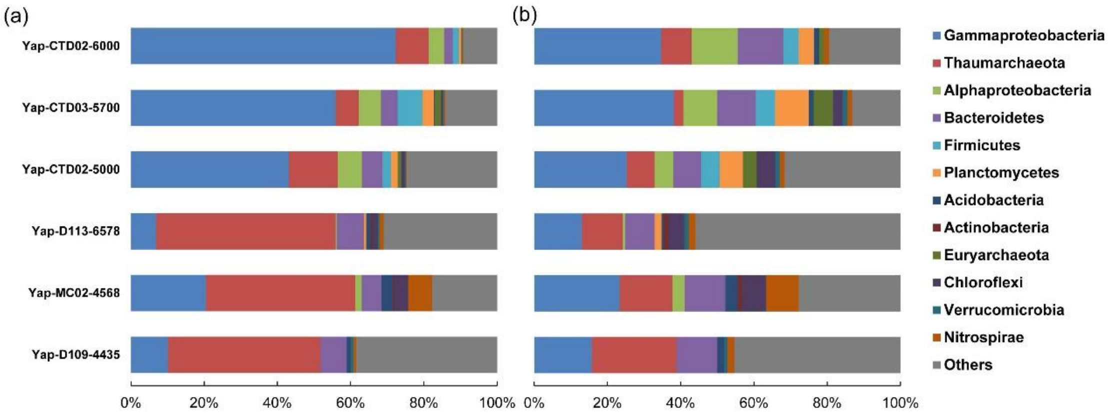

Figure S2. (a) Relative abundance and (b) diversity of the genomic bins identified in the 6 metagenomes of the Yap Trench. Abundance indicates the number of reads that mapped to a genomic bin, which normalized to the length of the genes and total number of reads in metagenome. Diversity indicates the number of genomic bins in each taxon.

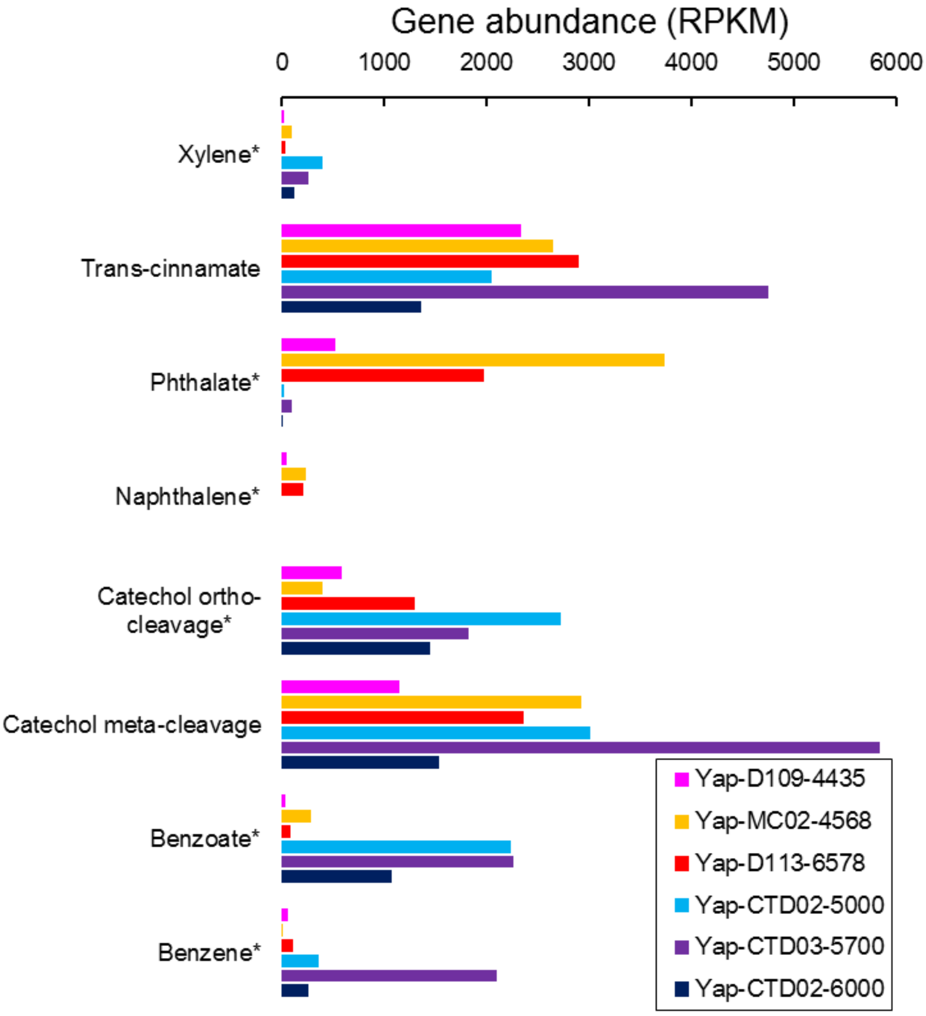

Figure S3. The abundance of genes involved in various aromatics degradation pathways in the 6 metagenomes of the Yap Trench. The asterisks indicate statistical significant between the 3 sediment and the 3 seawater metagenomes ($P<0.05$).

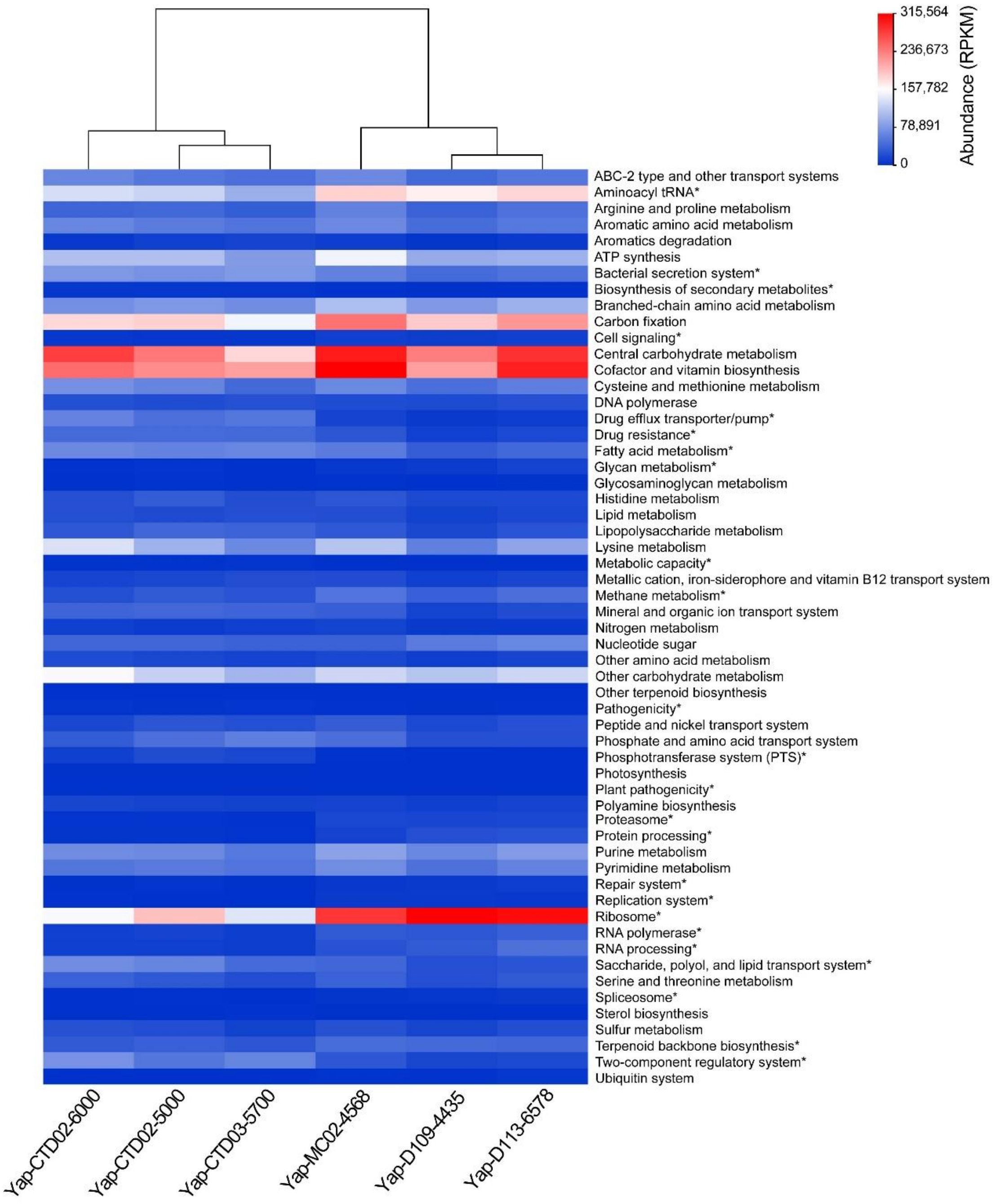

Figure S4. Heatmap showing the gene abundances involved in various metabolic pathways in the 6 metagenomes of the Yap Trench (according to KEGG category). The hierarchical cluster dendrogram is based on Bray-Curtis distance matrix. The asterisks indicate statistical significant between the 3 sediment and the 3 seawater metagenomes ($P<0.05$).

Table S1. Relative abundance of the microbial groups in each sample for generating Figures 1a and 1b.

Taxon / Sample	Gammaproteobacteria	Thaumarchaeota	Alphaproteobacteria	Bacteroidetes	Deferribacteres	Firmicutes	Deltaproteobacteria	Planctomycetes	Acidobacteria	Actinobacteria	Euryarchaeota	Gemmatimonadetes	Chloroflexi	Betaproteobacteria	Verrucomicrobia	Nitrospirae	Epsilonproteobacteria	Chlamydiae	Thermus-Deinococcus	Crenarchaeota	Others
Yap-109-4435	0.106	0.601	0.027	0.034	0.000	0.034	0.057	0.010	0.006	0.016	0.034	0.000	0.006	0.009	0.004	0.004	0.004	0.017	0.003	0.001	0.006
Yap-M02-4568	0.248	0.319	0.091	0.031	0.000	0.054	0.071	0.032	0.015	0.030	0.008	0.001	0.016	0.020	0.006	0.015	0.003	0.011	0.004	0.000	0.007
Yap-113-6578	0.152	0.531	0.033	0.022	0.000	0.046	0.058	0.016	0.012	0.019	0.031	0.000	0.012	0.012	0.003	0.005	0.004	0.008	0.005	0.001	0.008
Yap-5000	0.792	0.014	0.018	0.017	0.000	0.139	0.006	0.003	0.001	0.003	0.004	0.000	0.001	0.001	0.002	0.001	0.000	0.000	0.000	0.000	0.001
Yap-5700	0.812	0.005	0.049	0.007	0.000	0.106	0.004	0.004	0.001	0.002	0.004	0.000	0.002	0.001	0.002	0.001	0.000	0.000	0.001	0.000	0.001
Yap-6000	0.922	0.008	0.011	0.005	0.000	0.046	0.002	0.001	0.000	0.001	0.001	0.000	0.001	0.001	0.000	0.000	0.000	0.000	0.000	0.000	0.001
PRT-6000	0.196	0.000	0.405	0.038	0.076	0.002	0.046	0.032	0.019	0.013	0.033	0.011	0.031	0.023	0.022	0.000	0.000	0.002	0.000	0.012	0.015
MVD-4908	0.967	0.000	0.007	0.000	0.000	0.000	0.000	0.000	0.000	0.000	0.000	0.000	0.000	0.005	0.003	0.000	0.000	0.000	0.000	0.000	0.018
Mariana-5000	0.055	0.663	0.067	0.002	0.069	0.000	0.027	0.042	0.008	0.007	0.001	0.015	0.031	0.000	0.003	0.002	0.000	0.000	0.000	0.000	0.007
Mariana-6000	0.057	0.467	0.062	0.182	0.066	0.000	0.012	0.088	0.009	0.005	0.001	0.030	0.004	0.003	0.001	0.002	0.000	0.000	0.000	0.000	0.010
Mariana-7000	0.040	0.200	0.138	0.221	0.198	0.000	0.021	0.113	0.009	0.001	0.001	0.026	0.007	0.004	0.001	0.007	0.000	0.000	0.000	0.000	0.014
Mariana-7998	0.017	0.294	0.076	0.245	0.221	0.000	0.008	0.103	0.002	0.001	0.001	0.014	0.006	0.002	0.000	0.003	0.000	0.000	0.000	0.000	0.007
Mariana-9000	0.216	0.260	0.066	0.126	0.190	0.000	0.011	0.071	0.007	0.001	0.001	0.028	0.003	0.003	0.000	0.002	0.000	0.000	0.000	0.000	0.015
Mariana-10257	0.628	0.098	0.040	0.066	0.085	0.000	0.009	0.046	0.005	0.001	0.001	0.009	0.004	0.001	0.000	0.002	0.000	0.000	0.000	0.000	0.006
Japan-4989	0.165	0.308	0.033	0.077	0.033	0.000	0.121	0.066	0.044	0.022	0.022	0.022	0.022	0.000	0.011	0.011	0.000	0.000	0.000	0.000	0.044
Japan-5487	0.110	0.220	0.195	0.171	0.110	0.000	0.049	0.073	0.000	0.000	0.037	0.012	0.012	0.000	0.000	0.000	0.000	0.000	0.000	0.000	0.012
Japan-5988	0.108	0.301	0.097	0.140	0.065	0.000	0.065	0.097	0.032	0.000	0.032	0.032	0.022	0.000	0.000	0.000	0.000	0.000	0.000	0.000	0.011
Japan-6486	0.188	0.141	0.188	0.129	0.106	0.000	0.071	0.047	0.012	0.000	0.012	0.024	0.012	0.000	0.000	0.000	0.012	0.000	0.000	0.000	0.059
Japan-6986	0.177	0.165	0.153	0.165	0.071	0.000	0.082	0.094	0.024	0.012	0.012	0.012	0.012	0.000	0.012	0.000	0.000	0.000	0.000	0.000	0.012
Japan-7407	0.223	0.188	0.107	0.205	0.045	0.000	0.036	0.089	0.009	0.000	0.027	0.009	0.027	0.000	0.000	0.000	0.018	0.000	0.000	0.000	0.018

The code in *R* for the NMDS plot is “library("vegan")----otu <- read.table("input.txt", header=T)----row.names(otu) <- otu[,1]--otu <- otu[,-1]----nmds <- metaMDS(otu, distance = "bray", k=2, trymax=20)----plot(nmds, display="sites", choices=c(1, 2), type="t")”.

Table S2. Detailed information of the gene abundances involved in the microbial degradation pathways of carbohydrates, hydrocarbons, and aromatics as shown in Figures 1c, 1d, and S3.

Pathway	Gene description	Gene abundance (RPKM)					
		**Yap-D109-4435**	**Yap-MC02-4568**	**Yap-D113-6578**	**Yap-CTD02-5000**	**Yap-CTD03-5700**	**Yap-CTD02-6000**
Carbohydrates	Auxiliary activities (AAs)	3686.7	11405.5	5591.5	12981.7	26945.0	23440.5
	Carbohydrate-binding modules (CBMs)	27505.2	33444.3	34648.6	44028.0	29500.4	55733.5
	Carbohydrate esterases (CEs)	20652.8	32460.2	31431.1	47067.6	48138.9	46150.5
	Cellulosome	1399.6	897.3	2109.9	4237.6	2418.4	1994.0
	Glycoside hydrolases (GHs)	36827.3	53156.5	48313.8	83770.9	64469.6	76757.9
	Glycosyltransferases (GTs)	136291.7	94436.7	161304.4	104708.7	81375.6	88569.8
	Polysaccharide lyases (PLs)	2680.7	3972.1	5575.1	5617.7	4223.5	12320.0
Hydrocarbons	Alkane 1-monooxygenase *alkB*	43.6	191.8	113.3	971.6	4144.0	678.9
	7α-hydroxy-3-oxo-Δ4-cholenoic acid oxidoreductase *baiCD*	18.9	69.2	33.8	562.2	334.9	739.6
	Benzoate 1,2-dioxygenase hydroxylase *benA*	20.9	126.8	98.6	1472.2	849.7	516.3
	Biphenyl 2,3-dioxygenase *bph*	68.9	478.8	338.9	251.6	491.4	189.2
	Choloylglycine hydrolase *bsh*	0.0	0.0	6.9	696.8	192.8	208.2
	Carbazole 1,9a-dioxygenase *carA*	283.9	805.4	833.5	768.3	374.1	215.7
	Carnitine monooxygenase *cntA*	44.3	397.0	134.0	1007.4	450.2	377.2
	Choline trimethylamine-lyase *cutC*	10.6	0.0	0.0	302.3	340.8	232.8
	Dibenzofuran dioxygenase *dxnA/dbfA1*	9.4	160.0	107.9	19.6	59.1	10.1
	2-hydroxypropyl-CoM lyase *etnE*	6.5	22.4	36.8	435.8	343.3	148.4
	Cytochrome P450	106.6	175.9	172.9	565.0	752.6	248.4
	Vinyl chloride reductase *vcrA*	10.3	43.2	42.9	123.9	93.5	25.6
	Xenobiotic reductase A *xenA*	4.6	70.9	6.7	1654.9	346.2	3713.3
	Xenobiotic reductase B *xenB*	105.7	525.1	147.0	2648.5	4065.8	2487.0

Aromatics	Benzene	61.3	8.3	106.8	365.6	2096.2	257.6
	Benzoate	35.5	293.3	82.7	2239.2	2261.0	1076.4
	Catechol meta-cleavage	1151.3	2925.2	2359.2	3019.4	5842.2	1539.5
	Catechol ortho-cleavage	591.5	404.1	1300.9	2723.6	1829.3	1446.6
	Naphthalene	54.2	242.5	211.6	0.0	0.0	0.0
	Phthalate	520.8	3738.3	1969.9	27.6	95.1	2.7
	Trans-cinnamate	2337.9	2652.0	2901.5	2050.2	4750.2	1368.8
	Xylene	21.9	104.5	36.5	398.1	265.7	125.6

Table S3. Detailed information of the gene abundances involved in carbon fixation and nitrogen metabolisms as shown in Figure 2.

Pathway	Gene description	Gene abundance (RPKM)					
		Yap-D109-4435	Yap-MC02-4568	Yap-D113-6578	Yap-CTD02-5000	Yap-CTD03-5700	Yap-CTD02-6000
HP/HB cycle	Acetyl-CoA-propionyl-CoA carboxylase	3640.6	6083.3	6883.4	2427.4	1572.6	1446.4
	Malonyl-CoA reductase (NADPH)	2071.9	1970.5	2334.9	318.3	60.5	145.2
	3-hydroxypropionyl-CoA synthetase (ADP-forming)	1211.0	2526.9	1959.9	354.3	298.4	341.2
	3-hydroxypropionyl-CoA dehydratase	1717.5	3235.0	2567.4	3293.6	3210.7	2598.7
	Methylmalonyl-CoA epimerase	1646.1	2229.2	1465.2	396.8	340.6	534.3
	Methylmalonyl-CoA mutase	4201.1	5224.8	5240.4	2297.6	1711.0	1294.3
	4-hydroxybutyryl-CoA synthetase (ADP-forming)	1249.8	2032.5	2786.4	417.3	305.9	497.0
	4-hydroxybutyryl-CoA dehydratase	1087.4	1761.1	1118.7	748.1	1086.9	220.5
	3-hydroxybutyryl-CoA dehydrogenase	4725.8	4021.4	2768.6	892.3	1550.7	1844.5
	Propanoyl-CoA C-acyltransferase	4519.2	3629.0	3810.8	554.4	1475.6	338.6
Calvin cycle	Ribulose-bisphosphate carboxylase	181.1	560.1	256.1	128.0	40.1	25.3
	Phosphoglycerate kinase	5546.4	5500.6	6795.4	3110.5	1968.0	3892.8
	Glyceraldehyde-3-phosphate dehydrogenase	4103.7	4981.5	4950.3	4718.3	4379.7	6888.1
	Fructose-bisphosphate aldolase	897.3	1083.3	1043.8	4717.8	2439.9	2682.8
	Fructose-1,6-bisphosphatase	890.3	1431.3	1088.5	3313.1	1245.0	2119.7
	Transketolase	7187.8	6518.4	9770.0	5211.7	2935.6	3508.3
	Sedoheptulose-bisphosphatase	36.2	145.7	36.2	217.3	48.2	69.7
	Ribose 5-phosphate isomerase	3117.4	5869.7	3635.4	2928.7	1758.5	1505.4
	Phosphoribulokinase	170.1	702.4	287.8	466.8	657.4	984.1
Ammonification	Nitrogenase *nifH*	479.0	272.8	133.6	29.9	240.2	63.2
	Nitrite reductase *nirB*	1440.9	2598.3	1400.8	5907.3	7661.1	7625.5
	Nitrite reductase *nrfA*	25.9	237.1	51.0	104.0	110.2	76.8

	Periplasmic nitrate reductase *napA*	214.8	1009.5	310.5	2403.1	2392.3	2779.4
	Nitrate reductase *narG*	19.6	159.7	85.8	892.1	958.8	663.3
	Archaeal ammonia monooxygenase A_*amoA*	515.1	555.3	421.8	86.9	98.2	117.2
	Bacterial ammonia monooxygenase B_*amoA*	28.8	0.0	27.5	0.0	5.7	0.0
	Ammonia monooxygenase *amoB*	468.9	1654.6	780.3	219.1	98.2	158.8
Nitrification	Hydroxylamine oxidoreductase *hao*	69.4	408.8	238.5	34.0	27.7	27.5
	Nitrite oxidoreductase *nxrA*	5.6	209.9	64.5	7.7	4.0	0.0
	Nitrite oxidoreductase *nxrB*	12.0	246.6	84.2	354.7	543.9	258.2
	Nitrite oxidoreductase *nxrC*	273.0	1741.8	730.0	826.0	555.0	158.3
	Nitrite reductase *nirK*	2568.0	3941.4	3903.1	861.8	1131.8	3389.9
	Nitrite reductase *nirS*	0.0	0.0	0.0	0.0	20.3	108.2
Denitrification	Nitric oxide reductase *norB*	292.3	823.3	216.9	1746.1	2077.9	5130.1
	Nitric oxide reductase *norC*	9.4	65.0	41.2	11.5	70.9	127.6
	Nitrous oxide reductase *nosZ*	452.0	59.1	504.6	58.3	217.6	120.0
Anammox	Hydrazine synthase *hzsC*	0.0	0.0	23.48	0.0	0.0	0.0
	Hydrazine dehydrogenase *hdh*	11.5	0.0	0.0	0.0	0.0	0.0
Ammonia assimilation	Glutamine synthetase *glnA*	4772.4	9760.2	6502.3	8920.2	6920.8	5317.8
Ammonia mineralization	Urease *ureC*	1712.6	2167.2	2890.4	1181.9	711.5	688.9
	Alanine dehydrogenase *ald*	1405.1	2677.2	2083.6	3840.4	3341.2	2917.6

Table S4. Detailed information of the gene abundances involved in stress response and metal resistance pathways as shown in Figure 3.

Pathway	Gene description	Gene abundance (RPKM)					
		**Yap-D109-4435**	**Yap-MC02-4568**	**Yap-D113-6578**	**Yap-CTD02-5000**	**Yap-CTD03-5700**	**Yap-CTD02-6000**
Alkaline shock	Alkaline shock protein	0.0	0.0	5.0	64.6	19.8	8.7
Antioxidant enzyme	Catalase, alkyl hydroperoxide reductase, peroxiredoxin, superoxide dismutase, peroxidase	4025.9	2583.7	3893.2	5891.5	4917.5	4629.9
Cold shock	Cold shock protein cspA/cspB/cspG, Sensor histidine kinase desK, transcriptional regulatory protein DesR	4121.4	8068.4	4737.9	6959.5	7909.0	8799.9
Envelope stress	DNA-binding transcriptional regulator baeR/cpxR, sensory transduction histidine kinase, envelope stress induced periplasmic protein, phage shock protein A/B/C, sigma 54-dependent transcriptional activator	5635.4	8809.9	7197.4	17165.3	18650.7	19163.3
Heat shock	ATP-dependent Clp protease, chaperones DnaK/GroEL/GrpE/Cpn10, heat shock repressor HrcA, catalase,	9557.1	16282.3	13634.3	17512.8	13611.8	19046.6
Nitrogen limitation	Glutamine synthetase, glutamine synthetase repressor, nitrogen regulation protein NtrB/NtrC, transcription regulator TnrA	9121.6	23321.0	14607.8	12407.7	16396.4	14224.5
Osmotic stress	Sensor protein degS, DNA-binding response regulator DegU/KdpE/MtrA/ompR, sensor histidine kinase MtrB, Osmoregulated proline transporter, Osmoprotection protein proV/proW/proX	33692.5	64608.2	44960.0	59776.5	64231.6	78959.9
Oxidative stress	Alkyl hydroperoxide reductase, catalase, hydrogen peroxide-inducible genes activator OxyR, peroxide stress regulator PerR, superoxide dismutase, redox-sensitive transcriptional activator SoxR, regulatory protein SoxS	12890.1	21741.9	17433.2	21243.5	20886.0	29650.9
Oxygen limitation	Aerobic respiration control protein ArcA/ArcB, cytochrome d oxidase cyd subunit I/II, Crp/FNR family transcriptional regulator, nitrate reductase narH/narI/narJ, nitrite-sensitive transcriptional repressor NsrR, transcriptional regulatory protein ResD, sensor protein ResE	14345.9	22989.7	19114.4	27591.8	38809.7	56151.8
Phosphate limitation	Alkaline phosphatase, phosphate regulon transcriptional regulatory protein PhoB, phosphate ABC transporter, inner membrane subunit pstA/pstB/pstC/pstS	9539.8	15996.2	12915.0	15240.9	18889.2	29489.6

Stringent response	GTPase ObgE, (p)ppGpp synthetase SpoT/RelA	5929.7	8718.2	7664.5	9163.3	9031.4	8333.1
Al	7-cyano-7-deazaguanine synthase QueC	0.0	0.0	0.0	61.3	54.6	0.0
As/Sb	Thioredoxin-disulfide reductase, arsenical resistance protein ArsH, phosphate ABC transporter, ATP-binding protein pstA/pstB/pstC/pstS, arsenical resistance operon transcriptional repressor ArsD, transcriptional regulator arsR, arsenate reductase (thioredoxin), arsenical-resistance protein, arsenical pump-driving ATPase, arsenical efflux pump membrane protein ArsB	8.6	0.0	27.4	3143.9	2199.6	1754.6
Cd	Cd(II)/Pb(II)-responsive transcriptional regulator, ychH/yhcN, yodD	0.0	0.0	0.0	493.6	445.8	299.0
Cr	Oxygen-insensitive NADPH nitroreductase nfsA, chromate transporter, oscA	0.0	0.0	0.0	662.3	351.4	217.9
Co/Mg	Magnesium/cobalt transporter CorC, magnesium-translocating P-type ATPase, magnesium/cobalt efflux protein corB/corD	0.0	0.0	0.0	886.8	895.3	484.0
Cu	AsnC family transcriptional regulator copR, copper resistance protein CopB, divalent cation tolerance protein CutA, copY/tcrY, comR/ycfQ, dsbC, cutE/lnt, bhsA/ycfr/comC, cutC, copF, cutF/nlpE, cueR/ybbI, copZ, csoR	167.2	303.2	94.0	2267.8	3606.6	1651.0
Cu/Ag	Copper-exporting P-type ATPase CopA, cusR/ylcA, cusC/ylcB, cation efflux system protein CusB, cueA	0.0	28.3	6.7	1244.9	951.3	698.1
Cu/Co	Multicopper oxidase mco	0.0	0.0	0.0	956.6	329.5	254.8
Cu/Au	Copper-translocating P-type ATPase, golT	0.0	0.0	20.7	843.9	765.7	259.6
Fe	Histidine kinase PmrB, fetA/ybbL, fetB/ybbM, dpsA	74.5	218.8	206.8	490.4	437.4	318.4
Fe/Mn	Manganese/iron transporter ATP-binding protein sitA/sitB/sitC/sitD	0.0	0.0	0.0	677.5	632.1	463.1
Mn/Mg	Transcriptional regulator MntR, manganese efflux pump MntP	0.0	0.0	0.0	335.7	160.7	202.7
Mo/W	Molybdate ABC transporter substrate-binding protein modA/modB/modC/modE	0.0	0.0	0.0	583.0	399.6	291.2
Hg	Mercuric ion transporter merP, Hg(II)-responsive transcriptional regulator merR, merT, merF, merA, merB	14.7	70.7	22.7	2532.3	1535.8	1754.0
Ni	Nickel-responsive transcriptional regulator NikR, metal resistance protein ncrB	0.0	0.0	0.0	212.2	298.2	160.3
Ni/Co	MFS transporter nrsD/nreB, iron-dicitrate transporter subunit FecD, ferric citrate ABC transporter ATP-binding protein FecE	0.0	0.0	10.8	975.3	835.2	532.2

Se	Superoxide dismutase Se	30.0	54.6	11.0	670.3	306.3	225.5
Zn	Zinc ABC transporter substrate-binding protein znuA/yebL, transcriptional regulator Zur, multidrug resistance protein MdtC, mdtA, zinc transporter ZitB, Zn(2+)-responsive transcriptional regulator, two-component system response regulator ZraR	0.0	0.0	0.0	1804.4	1319.5	1005.4
Zn/Te	Phosphate transporter PitA	0.0	0.0	0.0	200.5	222.0	123.6
Zn/W	Two-component system response regulator BaeR/BaeS	0.0	0.0	0.0	428.4	219.5	194.6

Table S5. Detailed information of the genomic bins retrieved from the 6 metagenomes of the Yap Trench.

Genomic bin ID	Completeness (%)	Contamination (%)	RPKM	Number of contigs	Taxonomy		
					Domain	Phylum	Class
Yap-D109-4435_bin1	66.7	4.9	1.2	190	Archaea	Thaumarchaeota	Nitrososphaeria
Yap-D109-4435_bin2	45.2	12.6	5.5	179	Archaea	Thaumarchaeota	Nitrososphaeria
Yap-D109-4435_bin3	40.9	6.8	19.9	272	Archaea	Thaumarchaeota	Nitrososphaeria
Yap-D109-4435_bin4	38.4	3.6	3.5	110	Archaea	Thaumarchaeota	Nitrososphaeria
Yap-D109-4435_bin5	35.3	3.9	8.8	179	Archaea	Thaumarchaeota	Nitrososphaeria
Yap-D109-4435_bin6	32.6	8.4	1.4	74	Archaea	Nanoarchaeota	Woesearchaeia
Yap-D109-4435_bin7	31.3	5.7	1.9	132	Archaea	Nanoarchaeota	Woesearchaeia
Yap-D109-4435_bin8	27.9	0.0	1.2	72	Archaea	Thaumarchaeota	Nitrososphaeria
Yap-D109-4435_bin9	27.7	14.6	3.8	260	Archaea	Thaumarchaeota	Nitrososphaeria
Yap-D109-4435_bin10	27.6	7.3	4.0	144	Archaea	Thaumarchaeota	Nitrososphaeria
Yap-D109-4435_bin11	25.1	12.6	5.1	134	Archaea	Thaumarchaeota	Nitrososphaeria
Yap-D109-4435_bin12	24.3	2.7	0.9	45	Archaea	Nanoarchaeota	Woesearchaeia
Yap-D109-4435_bin13	22.3	0.0	0.7	96	Archaea	Thaumarchaeota	Nitrososphaeria
Yap-D109-4435_bin14	21.9	0.0	3.4	76	Archaea	Thaumarchaeota	Nitrososphaeria
Yap-D109-4435_bin15	20.1	0.5	8.1	59	Archaea	Thaumarchaeota	Nitrososphaeria
Yap-D109-4435_bin16	20.1	2.5	1.8	111	Archaea	Crenarchaeota	unclassified
Yap-D109-4435_bin17	19.9	2.8	0.7	136	Archaea	Nanoarchaeota	Woesearchaeia
Yap-D109-4435_bin18	19.5	1.7	0.9	340	Archaea	Thaumarchaeota	Nitrososphaeria
Yap-D109-4435_bin19	17.7	0.0	3.0	69	Archaea	Thaumarchaeota	Nitrososphaeria
Yap-D109-4435_bin20	17.7	4.9	1.5	127	Archaea	Thaumarchaeota	Nitrososphaeria
Yap-D109-4435_bin21	16.5	1.9	2.6	138	Archaea	Thaumarchaeota	Nitrososphaeria
Yap-D109-4435_bin22	12.6	1.3	2.2	73	Archaea	Thaumarchaeota	Nitrososphaeria
Yap-D109-4435_bin23	12.6	1.9	1.6	127	Archaea	Thaumarchaeota	Nitrososphaeria

Yap-D109-4435_bin24	11.8	0.0	0.8	65	Archaea	unclassified	unclassified
Yap-D109-4435_bin25	11.3	2.9	0.6	509	Archaea	Thaumarchaeota	Nitrososphaeria
Yap-D109-4435_bin26	10.8	1.0	6.3	91	Archaea	Thaumarchaeota	Nitrososphaeria
Yap-D109-4435_bin27	10.6	1.0	1.0	48	Archaea	Thaumarchaeota	Nitrososphaeria
Yap-D109-4435_bin28	10.2	0.0	0.8	65	Archaea	Thaumarchaeota	Nitrososphaeria
Yap-D109-4435_bin29	9.7	2.9	4.8	93	Archaea	Thaumarchaeota	Nitrososphaeria
Yap-D109-4435_bin30	9.4	0.0	1.3	64	Archaea	Nanoarchaeota	Woesearchaeia
Yap-D109-4435_bin31	8.7	1.0	0.6	60	Archaea	Thaumarchaeota	Nitrososphaeria
Yap-D109-4435_bin32	91.6	7.4	4.9	206	Bacteria	Dadabacteria	UBA1144
Yap-D109-4435_bin33	78.5	8.0	2.5	282	Bacteria	Proteobacteria	Gammaproteobacteria
Yap-D109-4435_bin34	76.6	4.9	7.2	266	Bacteria	Dadabacteria	UBA1144
Yap-D109-4435_bin35	75.2	11.8	1.9	379	Bacteria	Proteobacteria	Gammaproteobacteria
Yap-D109-4435_bin36	74.4	18.8	3.1	518	Bacteria	Dadabacteria	UBA1144
Yap-D109-4435_bin37	73.8	18.2	2.4	753	Bacteria	Bacteroidetes	Ignavibacteria
Yap-D109-4435_bin38	70.6	9.8	1.2	621	Bacteria	Bacteroidetes	Bacteroidia
Yap-D109-4435_bin39	59.7	14.1	1.4	290	Bacteria	Acidobacteria	unclassified
Yap-D109-4435_bin40	57.6	6.3	1.4	328	Bacteria	Bacteroidetes	Bacteroidia
Yap-D109-4435_bin41	56.5	2.7	1.4	189	Bacteria	Proteobacteria	Gammaproteobacteria
Yap-D109-4435_bin42	55.5	1.7	2.4	195	Bacteria	Dadabacteria	UBA1144
Yap-D109-4435_bin43	55.0	3.8	1.0	451	Bacteria	Proteobacteria	Gammaproteobacteria
Yap-D109-4435_bin44	53.9	4.0	1.8	418	Bacteria	Dadabacteria	UBA1144
Yap-D109-4435_bin45	53.3	4.3	1.3	268	Bacteria	Omnitrophica	unclassified
Yap-D109-4435_bin46	51.9	28.3	1.3	152	Bacteria	Omnitrophica	koll11
Yap-D109-4435_bin47	51.5	9.7	2.7	194	Bacteria	Proteobacteria	Gammaproteobacteria
Yap-D109-4435_bin48	48.3	4.2	1.0	354	Bacteria	Nitrospirae	Nitrospiria
Yap-D109-4435_bin49	45.5	4.3	1.2	353	Bacteria	Nitrospinae	Nitrospinia

Yap-D109-4435_bin50	41.5	9.5	1.4	305	Bacteria	unclassified	unclassified
Yap-D109-4435_bin51	39.4	17.2	0.6	683	Bacteria	Proteobacteria	Gammaproteobacteria
Yap-D109-4435_bin52	36.2	6.9	1.0	250	Bacteria	Bacteroidetes	unclassified
Yap-D109-4435_bin53	34.4	3.4	4.3	376	Bacteria	Dadabacteria	UBA1144
Yap-D109-4435_bin54	32.9	0.0	3.2	48	Bacteria	UBP10	GR-WP33-30
Yap-D109-4435_bin55	32.0	4.1	1.0	261	Bacteria	unclassified	unclassified
Yap-D109-4435_bin56	29.2	7.3	0.7	192	Bacteria	Patescibacteria	Paceibacteria
Yap-D109-4435_bin57	25.6	3.9	1.1	92	Bacteria	Proteobacteria	Gammaproteobacteria
Yap-D109-4435_bin58	24.3	0.0	0.9	219	Bacteria	Acidobacteria	unclassified
Yap-D109-4435_bin59	24.1	1.7	0.8	113	Bacteria	Proteobacteria	Gammaproteobacteria
Yap-D109-4435_bin60	24.1	3.5	1.7	89	Bacteria	Bacteroidetes	Ignavibacteria
Yap-D109-4435_bin61	23.3	10.3	1.7	59	Bacteria	Patescibacteria	Paceibacteria
Yap-D109-4435_bin62	22.6	0.1	0.7	204	Bacteria	Bacteroidetes	unclassified
Yap-D109-4435_bin63	21.6	0.0	1.6	76	Bacteria	Verrucomicrobia	unclassified
Yap-D109-4435_bin64	21.3	0.3	1.7	203	Bacteria	Bacteroidetes	Bacteroidia
Yap-D109-4435_bin65	20.1	0.0	2.5	276	Bacteria	Dadabacteria	UBA1144
Yap-D109-4435_bin66	19.9	1.7	2.6	143	Bacteria	unclassified	unclassified
Yap-D109-4435_bin67	19.7	3.5	1.3	209	Bacteria	Dadabacteria	UBA1144
Yap-D109-4435_bin68	18.1	2.8	0.5	168	Bacteria	Omnitrophica	unclassified
Yap-D109-4435_bin69	17.4	3.5	1.7	133	Bacteria	Proteobacteria	Gammaproteobacteria
Yap-D109-4435_bin70	17.2	0.0	1.2	76	Bacteria	Proteobacteria	Gammaproteobacteria
Yap-D109-4435_bin71	17.1	1.7	1.2	164	Bacteria	Nitrospinae	Nitrospinia
Yap-D109-4435_bin72	16.4	0.0	1.5	44	Bacteria	Proteobacteria	Gammaproteobacteria
Yap-D109-4435_bin73	16.1	0.8	0.8	456	Bacteria	Nitrospirae	Nitrospiria
Yap-D109-4435_bin74	15.7	0.0	0.7	162	Bacteria	unclassified	unclassified
Yap-D109-4435_bin75	15.5	4.3	0.7	85	Bacteria	Margulisbacteria	unclassified

Yap-D109-4435_bin76	15.4	0.0	1.1	43	Bacteria	Proteobacteria	Gammaproteobacteria
Yap-D109-4435_bin77	15.1	0.0	1.2	73	Bacteria	unclassified	unclassified
Yap-D109-4435_bin78	14.4	0.0	0.5	133	Bacteria	Dependentiae	Babeliae
Yap-D109-4435_bin79	13.5	0.4	0.9	116	Bacteria	Proteobacteria	Gammaproteobacteria
Yap-D109-4435_bin80	12.5	3.6	0.9	260	Bacteria	Proteobacteria	Gammaproteobacteria
Yap-D109-4435_bin81	12.4	0.0	2.3	208	Bacteria	unclassified	unclassified
Yap-D109-4435_bin82	12.2	0.0	2.7	30	Bacteria	unclassified	unclassified
Yap-D109-4435_bin83	11.7	0.4	1.3	42	Bacteria	Proteobacteria	Gammaproteobacteria
Yap-D109-4435_bin84	9.9	0.0	1.5	99	Bacteria	unclassified	unclassified
Yap-D109-4435_bin85	9.2	0.0	1.0	115	Bacteria	Bacteroidetes	Bacteroidia
Yap-D109-4435_bin86	9.2	1.7	1.2	48	Bacteria	Dependentiae	Babeliae
Yap-D109-4435_bin87	8.9	1.7	1.2	255	Bacteria	unclassified	unclassified
Yap-D109-4435_bin88	8.7	0.3	1.2	42	Bacteria	Bacteroidetes	Bacteroidia
Yap-D109-4435_bin89	8.6	0.0	1.0	26	Bacteria	Proteobacteria	Gammaproteobacteria
Yap-D109-4435_bin90	8.5	0.0	0.8	105	Bacteria	Zixibacteria	MSB-5A5
Yap-D109-4435_bin91	8.2	0.0	0.9	131	Bacteria	Nitrospirae	Nitrospiria
Yap-D109-4435_bin92	8.0	0.0	1.8	35	Bacteria	Bacteroidetes	Ignavibacteria
Yap-D109-4435_bin93	7.0	0.0	9.3	136	Bacteria	Dadabacteria	UBA1144
Yap-D109-4435_bin94	6.2	0.1	0.7	81	Bacteria	Proteobacteria	unclassified
Yap-D109-4435_bin95	6.0	0.0	0.7	87	Bacteria	Omnitrophica	Omnitrophica
Yap-D109-4435_bin96	6.0	0.0	1.0	38	Bacteria	Bacteroidetes	Bacteroidia
Yap-D109-4435_bin97	5.6	0.0	0.6	84	Bacteria	unclassified	unclassified
Yap-D109-4435_bin98	4.4	0.0	0.6	41	Bacteria	unclassified	unclassified
Yap-D109-4435_bin99	3.5	0.0	1.0	30	Bacteria	Proteobacteria	Gammaproteobacteria
Yap-D109-4435_bin100	3.0	0.0	1.6	33	Bacteria	unclassified	unclassified
Yap-D109-4435_bin101	1.7	0.0	0.9	178	Bacteria	Bacteroidetes	Bacteroidia

Yap-D109-4435_bin102	0.8	0.0	0.7	43	Bacteria	Patescibacteria	Paceibacteria
Yap-D109-4435_bin103	24.2	1.9	0.6	1491	unclassified	unclassified	unclassified
Yap-D109-4435_bin104	22.7	1.0	1.3	154	unclassified	unclassified	unclassified
Yap-D109-4435_bin105	10.3	0.0	1.6	6043	unclassified	unclassified	unclassified
Yap-D109-4435_bin106	8.3	0.0	1.0	2917	unclassified	unclassified	unclassified
Yap-D109-4435_bin107	6.1	0.2	1.8	91	unclassified	unclassified	unclassified
Yap-D109-4435_bin108	5.6	0.0	1.2	1312	unclassified	unclassified	unclassified
Yap-D109-4435_bin109	0.0	0.0	0.7	3225	unclassified	unclassified	unclassified
Yap-MC02-4568_bin1	52.4	5.8	27.5	113	Archaea	Thaumarchaeota	Nitrososphaeria
Yap-MC02-4568_bin2	38.7	2.9	1.5	145	Archaea	Thaumarchaeota	Nitrososphaeria
Yap-MC02-4568_bin3	38.6	4.9	1.7	285	Archaea	Thaumarchaeota	Nitrososphaeria
Yap-MC02-4568_bin4	34.6	5.8	25.0	205	Archaea	Thaumarchaeota	Nitrososphaeria
Yap-MC02-4568_bin5	32.8	0.0	1.7	145	Archaea	Crenarchaeota	unclassified
Yap-MC02-4568_bin6	27.0	4.2	4.9	62	Archaea	Thaumarchaeota	Nitrososphaeria
Yap-MC02-4568_bin7	27.0	0.0	1.3	86	Archaea	Nanoarchaeota	Woesearchaeia
Yap-MC02-4568_bin8	22.3	8.7	2.2	713	Archaea	Thaumarchaeota	Nitrososphaeria
Yap-MC02-4568_bin9	19.3	1.9	23.6	124	Archaea	Thaumarchaeota	Nitrososphaeria
Yap-MC02-4568_bin10	11.7	1.9	10.5	77	Archaea	Thaumarchaeota	Nitrososphaeria
Yap-MC02-4568_bin11	9.0	0.3	1.7	67	Archaea	Thaumarchaeota	Nitrososphaeria
Yap-MC02-4568_bin12	7.2	0.0	2.2	81	Archaea	Thaumarchaeota	Nitrososphaeria
Yap-MC02-4568_bin13	6.1	2.4	2.4	82	Archaea	Thaumarchaeota	Nitrososphaeria
Yap-MC02-4568_bin14	4.2	0.0	48.2	46	Archaea	Thaumarchaeota	Nitrososphaeria
Yap-MC02-4568_bin15	92.2	1.3	3.3	141	Bacteria	Proteobacteria	Gammaproteobacteria
Yap-MC02-4568_bin16	92.2	13.7	3.0	459	Bacteria	Bacteroidetes	Bacteroidia
Yap-MC02-4568_bin17	87.9	11.3	2.2	796	Bacteria	Nitrospirae	Nitrospiria
Yap-MC02-4568_bin18	84.6	37.9	1.7	1261	Bacteria	Bacteroidetes	Bacteroidia

Yap-MC02-4568_bin19	83.1	4.1	2.4	585	Bacteria	Proteobacteria	Alphaproteobacteria
Yap-MC02-4568_bin20	80.9	16.8	3.4	624	Bacteria	Chloroflexi	Anaerolineae
Yap-MC02-4568_bin21	76.3	7.2	3.9	238	Bacteria	Proteobacteria	Gammaproteobacteria
Yap-MC02-4568_bin22	73.7	6.2	5.4	198	Bacteria	Proteobacteria	Gammaproteobacteria
Yap-MC02-4568_bin23	73.5	6.4	5.2	363	Bacteria	Dadabacteria	UBA1144
Yap-MC02-4568_bin24	73.5	7.7	3.7	499	Bacteria	Nitrospirae	Nitrospiria
Yap-MC02-4568_bin25	72.7	6.0	2.1	294	Bacteria	Proteobacteria	Gammaproteobacteria
Yap-MC02-4568_bin26	65.2	3.1	10.8	132	Bacteria	Proteobacteria	Gammaproteobacteria
Yap-MC02-4568_bin27	62.7	22.8	1.8	734	Bacteria	Bacteroidetes	Bacteroidia
Yap-MC02-4568_bin28	62.7	5.3	3.7	202	Bacteria	Dadabacteria	UBA1144
Yap-MC02-4568_bin29	60.1	5.9	2.2	387	Bacteria	Chloroflexi	Dehalococcoidia
Yap-MC02-4568_bin30	59.3	19.8	1.8	859	Bacteria	Acidobacteria	unclassified
Yap-MC02-4568_bin31	58.7	1.2	1.9	357	Bacteria	Chloroflexi	Dehalococcoidia
Yap-MC02-4568_bin32	55.2	2.6	2.7	409	Bacteria	Proteobacteria	Gammaproteobacteria
Yap-MC02-4568_bin33	54.9	17.6	5.0	690	Bacteria	Dadabacteria	UBA1144
Yap-MC02-4568_bin34	52.5	15.5	3.3	587	Bacteria	Bacteroidetes	Ignavibacteria
Yap-MC02-4568_bin35	51.8	6.6	5.2	450	Bacteria	Proteobacteria	Gammaproteobacteria
Yap-MC02-4568_bin36	51.0	1.3	1.7	291	Bacteria	Proteobacteria	Gammaproteobacteria
Yap-MC02-4568_bin37	50.6	11.7	3.0	278	Bacteria	Proteobacteria	Gammaproteobacteria
Yap-MC02-4568_bin38	50.4	3.0	2.0	778	Bacteria	Chloroflexi	Dehalococcoidia
Yap-MC02-4568_bin39	47.7	2.9	5.5	394	Bacteria	Nitrospirae	Nitrospiria
Yap-MC02-4568_bin40	45.8	4.0	2.7	485	Bacteria	Bacteroidetes	Ignavibacteria
Yap-MC02-4568_bin41	43.0	1.7	1.6	365	Bacteria	Proteobacteria	Gammaproteobacteria
Yap-MC02-4568_bin42	42.5	5.2	1.6	322	Bacteria	Dadabacteria	UBA1144
Yap-MC02-4568_bin43	42.3	6.3	7.7	431	Bacteria	UBP10	GR-WP33-30
Yap-MC02-4568_bin44	41.8	1.9	1.7	251	Bacteria	Dependentiae	Babeliae

Yap-MC02-4568_bin45	39.0	0.0	1.7	295	Bacteria	Nitrospinae	Nitrospinia
Yap-MC02-4568_bin46	37.2	3.6	4.0	273	Bacteria	UBP10	GR-WP33-30
Yap-MC02-4568_bin47	36.2	0.0	3.5	395	Bacteria	Proteobacteria	Gammaproteobacteria
Yap-MC02-4568_bin48	32.8	0.9	1.9	198	Bacteria	Proteobacteria	Gammaproteobacteria
Yap-MC02-4568_bin49	31.8	1.7	1.4	406	Bacteria	Proteobacteria	Gammaproteobacteria
Yap-MC02-4568_bin50	30.3	2.5	6.6	229	Bacteria	Proteobacteria	Gammaproteobacteria
Yap-MC02-4568_bin51	29.6	16.7	1.7	549	Bacteria	Nitrospinae	Nitrospinia
Yap-MC02-4568_bin52	28.8	0.9	1.7	125	Bacteria	Nitrospirae	Nitrospiria
Yap-MC02-4568_bin53	26.0	6.2	4.9	105	Bacteria	Acidobacteria	unclassified
Yap-MC02-4568_bin54	25.9	1.7	1.2	528	Bacteria	Bacteroidetes	unclassified
Yap-MC02-4568_bin55	24.1	0.0	3.1	69	Bacteria	Nitrospinae	Nitrospinia
Yap-MC02-4568_bin56	24.1	3.0	7.5	273	Bacteria	Proteobacteria	Gammaproteobacteria
Yap-MC02-4568_bin57	23.4	1.7	1.8	151	Bacteria	Nitrospinae	Nitrospinia
Yap-MC02-4568_bin58	22.4	1.9	3.8	228	Bacteria	UBP10	GR-WP33-30
Yap-MC02-4568_bin59	22.4	1.7	4.1	173	Bacteria	Proteobacteria	Gammaproteobacteria
Yap-MC02-4568_bin60	22.2	0.0	1.6	49	Bacteria	Actinobacteria	Acidimicrobiia
Yap-MC02-4568_bin61	21.7	0.6	1.6	190	Bacteria	Nitrospirae	Nitrospiria
Yap-MC02-4568_bin62	21.7	3.3	5.8	133	Bacteria	Proteobacteria	Gammaproteobacteria
Yap-MC02-4568_bin63	21.2	4.7	1.6	362	Bacteria	Nitrospirae	Nitrospiria
Yap-MC02-4568_bin64	20.7	0.0	1.6	156	Bacteria	Bacteroidetes	Bacteroidia
Yap-MC02-4568_bin65	20.7	0.9	1.6	179	Bacteria	Nitrospinae	Nitrospinia
Yap-MC02-4568_bin66	18.0	0.5	1.3	114	Bacteria	Desulfobacteraeota	unclassified
Yap-MC02-4568_bin67	17.7	0.0	1.0	410	Bacteria	Proteobacteria	Gammaproteobacteria
Yap-MC02-4568_bin68	16.6	0.0	6.8	47	Bacteria	Nitrospirae	Nitrospiria
Yap-MC02-4568_bin69	15.7	0.0	1.2	507	Bacteria	Bacteroidetes	unclassified
Yap-MC02-4568_bin70	15.4	1.8	1.4	100	Bacteria	Chloroflexi	Dehalococcoidia

Yap-MC02-4568_bin71	13.8	0.0	1.2	293	Bacteria	Proteobacteria	Gammaproteobacteria
Yap-MC02-4568_bin72	12.6	0.7	5.0	85	Bacteria	Acidobacteria	unclassified
Yap-MC02-4568_bin73	12.5	0.0	6.1	151	Bacteria	Nitrospinae	Nitrospinia
Yap-MC02-4568_bin74	12.2	0.0	1.5	129	Bacteria	Nitrospirae	Nitrospiria
Yap-MC02-4568_bin75	11.2	0.0	1.7	142	Bacteria	Bacteroidetes	Bacteroidia
Yap-MC02-4568_bin76	11.1	1.7	2.7	306	Bacteria	UBP10	unclassified
Yap-MC02-4568_bin77	9.9	1.8	1.9	140	Bacteria	Proteobacteria	Alphaproteobacteria
Yap-MC02-4568_bin78	9.3	0.0	1.1	139	Bacteria	unclassified	unclassified
Yap-MC02-4568_bin79	8.6	0.0	2.7	132	Bacteria	UBP10	GR-WP33-30
Yap-MC02-4568_bin80	8.6	0.0	1.0	66	Bacteria	Methylomirabilaeota	Methylomirabilia
Yap-MC02-4568_bin81	8.2	0.0	1.2	78	Bacteria	unclassified	unclassified
Yap-MC02-4568_bin82	5.8	3.5	2.1	130	Bacteria	Proteobacteria	Gammaproteobacteria
Yap-MC02-4568_bin83	5.7	0.0	1.3	134	Bacteria	Proteobacteria	unclassified
Yap-MC02-4568_bin84	5.1	0.0	3.0	293	Bacteria	Chloroflexi	Anaerolineae
Yap-MC02-4568_bin85	5.1	0.0	2.1	206	Bacteria	Proteobacteria	Gammaproteobacteria
Yap-MC02-4568_bin86	3.6	0.5	1.5	511	Bacteria	unclassified	unclassified
Yap-MC02-4568_bin87	2.8	0.0	2.4	23	Bacteria	Proteobacteria	Alphaproteobacteria
Yap-MC02-4568_bin88	2.7	0.0	2.2	97	Bacteria	Bacteroidetes	Bacteroidia
Yap-MC02-4568_bin89	5.5	0.0	2.1	48	unclassified	unclassified	unclassified
Yap-MC02-4568_bin90	2.4	0.0	1.7	233	unclassified	unclassified	unclassified
Yap-D113-6578_bin1	56.3	12.6	31.3	177	Archaea	Thaumarchaeota	Nitrososphaeria
Yap-D113-6578_bin2	55.3	11.8	9.8	238	Archaea	Thaumarchaeota	Nitrososphaeria
Yap-D113-6578_bin3	45.0	5.8	27.3	92	Archaea	Thaumarchaeota	Nitrososphaeria
Yap-D113-6578_bin4	37.5	12.7	8.0	132	Archaea	Thaumarchaeota	Nitrososphaeria
Yap-D113-6578_bin5	37.0	9.0	4.1	176	Archaea	Thaumarchaeota	Nitrososphaeria
Yap-D113-6578_bin6	36.0	1.0	1.0	78	Archaea	Thaumarchaeota	Nitrososphaeria

Yap-D113-6578_bin7	32.0	1.6	2.2	90	Archaea	Nanoarchaeota	Woesearchaeia
Yap-D113-6578_bin8	28.6	0.0	6.2	64	Archaea	Thaumarchaeota	Nitrososphaeria
Yap-D113-6578_bin9	28.2	6.5	2.5	78	Archaea	Nanoarchaeota	Woesearchaeia
Yap-D113-6578_bin10	25.0	0.9	3.0	73	Archaea	Nanoarchaeota	Woesearchaeia
Yap-D113-6578_bin11	24.6	2.3	3.4	59	Archaea	Thaumarchaeota	Nitrososphaeria
Yap-D113-6578_bin12	23.2	0.0	1.5	37	Archaea	Nanoarchaeota	Woesearchaeia
Yap-D113-6578_bin13	22.2	0.9	0.5	67	Archaea	Nanoarchaeota	Woesearchaeia
Yap-D113-6578_bin14	18.8	0.0	4.9	68	Archaea	Thaumarchaeota	Nitrososphaeria
Yap-D113-6578_bin15	18.5	1.2	4.1	70	Archaea	Thaumarchaeota	Nitrososphaeria
Yap-D113-6578_bin16	17.2	0.0	19.1	60	Archaea	Thaumarchaeota	Nitrososphaeria
Yap-D113-6578_bin17	16.8	0.9	1.5	23	Archaea	Nanoarchaeota	Woesearchaeia
Yap-D113-6578_bin18	16.5	2.9	1.5	264	Archaea	Thaumarchaeota	Nitrososphaeria
Yap-D113-6578_bin19	16.1	1.4	0.8	36	Archaea	Nanoarchaeota	Woesearchaeia
Yap-D113-6578_bin20	15.9	0.0	0.8	45	Archaea	Nanoarchaeota	Woesearchaeia
Yap-D113-6578_bin21	15.0	6.1	1.2	39	Archaea	Nanoarchaeota	Woesearchaeia
Yap-D113-6578_bin22	13.8	2.9	27.8	113	Archaea	Thaumarchaeota	Nitrososphaeria
Yap-D113-6578_bin23	13.6	0.9	1.2	31	Archaea	Microarchaeota	Iainarchaeia
Yap-D113-6578_bin24	13.1	0.0	1.1	40	Archaea	Nanoarchaeota	Woesearchaeia
Yap-D113-6578_bin25	12.9	0.9	0.6	37	Archaea	Nanoarchaeota	Woesearchaeia
Yap-D113-6578_bin26	11.7	1.3	0.8	79	Archaea	Nanoarchaeota	Woesearchaeia
Yap-D113-6578_bin27	11.3	0.0	1.6	51	Archaea	Thaumarchaeota	Nitrososphaeria
Yap-D113-6578_bin28	11.2	0.0	1.2	29	Archaea	Nanoarchaeota	Woesearchaeia
Yap-D113-6578_bin29	10.4	1.0	2.5	71	Archaea	Thaumarchaeota	Nitrososphaeria
Yap-D113-6578_bin30	10.2	1.5	2.0	61	Archaea	Thaumarchaeota	Nitrososphaeria
Yap-D113-6578_bin31	7.9	0.0	0.5	217	Archaea	Thaumarchaeota	Nitrososphaeria
Yap-D113-6578_bin32	7.5	0.9	1.8	24	Archaea	Nanoarchaeota	Woesearchaeia

Yap-D113-6578_bin33	6.8	0.0	0.7	32	Archaea	Nanoarchaeota	Woesearchaeia
Yap-D113-6578_bin34	6.4	1.9	2.2	103	Archaea	Thaumarchaeota	Nitrososphaeria
Yap-D113-6578_bin35	6.1	0.0	2.1	40	Archaea	Nanoarchaeota	Woesearchaeia
Yap-D113-6578_bin36	86.0	1.8	1.1	510	Bacteria	Proteobacteria	Gammaproteobacteria
Yap-D113-6578_bin37	75.9	13.7	1.2	386	Bacteria	Nitrospirae	Nitrospiria
Yap-D113-6578_bin38	71.5	8.0	6.2	330	Bacteria	UBP10	GR-WP33-30
Yap-D113-6578_bin39	64.5	3.6	1.6	52	Bacteria	Patescibacteria	WWE3
Yap-D113-6578_bin40	62.7	14.2	1.6	538	Bacteria	Nitrospinae	Nitrospinia
Yap-D113-6578_bin41	59.8	35.1	0.9	635	Bacteria	Omnitrophica	koll11
Yap-D113-6578_bin42	59.2	3.3	2.0	256	Bacteria	Bacteroidetes	unclassified
Yap-D113-6578_bin43	57.7	2.2	1.3	326	Bacteria	Proteobacteria	Gammaproteobacteria
Yap-D113-6578_bin44	57.2	10.2	3.1	557	Bacteria	Dadabacteria	UBA1144
Yap-D113-6578_bin45	56.1	20.5	0.7	736	Bacteria	Nitrospinae	Nitrospinia
Yap-D113-6578_bin46	55.2	15.6	0.8	635	Bacteria	UBP10	GR-WP33-30
Yap-D113-6578_bin47	53.9	7.8	3.5	297	Bacteria	Dadabacteria	UBA1144
Yap-D113-6578_bin48	53.5	5.1	1.3	531	Bacteria	Bacteroidetes	Bacteroidia
Yap-D113-6578_bin49	50.7	30.8	1.3	391	Bacteria	Marinimicrobia	unclassified
Yap-D113-6578_bin50	49.6	12.7	0.9	445	Bacteria	Elusimicrobia	unclassified
Yap-D113-6578_bin51	48.3	5.2	1.1	131	Bacteria	Proteobacteria	Gammaproteobacteria
Yap-D113-6578_bin52	47.7	6.4	1.2	382	Bacteria	Bacteroidetes	Bacteroidia
Yap-D113-6578_bin53	47.6	0.9	2.5	201	Bacteria	Nitrospinae	Nitrospinia
Yap-D113-6578_bin54	47.6	17.2	0.9	267	Bacteria	Verrucomicrobia	Chlamydiia
Yap-D113-6578_bin55	47.4	11.0	0.7	325	Bacteria	Omnitrophica	koll11
Yap-D113-6578_bin56	47.3	5.6	3.5	346	Bacteria	Bacteroidetes	Ignavibacteria
Yap-D113-6578_bin57	47.0	12.5	1.7	339	Bacteria	Proteobacteria	Gammaproteobacteria
Yap-D113-6578_bin58	44.6	0.0	0.6	220	Bacteria	Actinobacteria	Acidimicrobiia

Yap-D113-6578_bin59	44.5	5.5	2.1	181	Bacteria	Proteobacteria	Gammaproteobacteria
Yap-D113-6578_bin60	44.4	35.4	0.7	268	Bacteria	Dependentiae	Babeliae
Yap-D113-6578_bin61	44.3	12.1	0.9	90	Bacteria	Patescibacteria	Paceibacteria
Yap-D113-6578_bin62	43.9	26.0	0.6	1459	Bacteria	unclassified	unclassified
Yap-D113-6578_bin63	43.5	7.1	0.9	353	Bacteria	Proteobacteria	Gammaproteobacteria
Yap-D113-6578_bin64	43.1	12.1	0.8	120	Bacteria	Proteobacteria	Gammaproteobacteria
Yap-D113-6578_bin65	41.4	14.6	0.5	2555	Bacteria	unclassified	unclassified
Yap-D113-6578_bin66	40.0	9.0	0.6	143	Bacteria	Patescibacteria	Paceibacteria
Yap-D113-6578_bin67	40.0	3.7	4.0	265	Bacteria	Acidobacteria	unclassified
Yap-D113-6578_bin68	39.2	7.5	0.8	315	Bacteria	unclassified	unclassified
Yap-D113-6578_bin69	37.5	2.3	2.1	210	Bacteria	Proteobacteria	Gammaproteobacteria
Yap-D113-6578_bin70	37.3	10.3	0.6	235	Bacteria	Omnitrophica	koll11
Yap-D113-6578_bin71	37.3	5.0	1.4	506	Bacteria	unclassified	unclassified
Yap-D113-6578_bin72	36.8	1.1	0.8	126	Bacteria	Bdellovibrionaeota	UBA2394
Yap-D113-6578_bin73	36.1	6.9	0.7	208	Bacteria	Bacteroidetes	Ignavibacteria
Yap-D113-6578_bin74	35.2	5.5	1.9	290	Bacteria	Bacteroidetes	Bacteroidia
Yap-D113-6578_bin75	34.7	6.5	0.9	164	Bacteria	Proteobacteria	Gammaproteobacteria
Yap-D113-6578_bin76	34.6	3.5	1.2	74	Bacteria	Patescibacteria	Paceibacteria
Yap-D113-6578_bin77	34.5	10.3	1.1	552	Bacteria	Bacteroidetes	Bacteroidia
Yap-D113-6578_bin78	33.5	0.0	0.7	239	Bacteria	Chloroflexi	Anaerolineae
Yap-D113-6578_bin79	33.3	2.3	1.1	124	Bacteria	Omnitrophica	Omnitrophica
Yap-D113-6578_bin80	32.0	9.3	0.5	1584	Bacteria	Proteobacteria	Gammaproteobacteria
Yap-D113-6578_bin81	31.6	7.3	0.5	767	Bacteria	Chloroflexi	Dehalococcoidia
Yap-D113-6578_bin82	30.6	6.3	0.6	402	Bacteria	unclassified	unclassified
Yap-D113-6578_bin83	29.8	0.0	0.6	195	Bacteria	unclassified	unclassified
Yap-D113-6578_bin84	29.3	5.0	0.5	116	Bacteria	Patescibacteria	Paceibacteria

Yap-D113-6578_bin85	29.2	1.4	0.7	157	Bacteria	Verrucomicrobia	Verrucomicrobiae
Yap-D113-6578_bin86	28.7	0.1	1.1	112	Bacteria	Bacteroidetes	Bacteroidia
Yap-D113-6578_bin87	28.5	0.9	2.0	176	Bacteria	Nitrospinae	Nitrospinia
Yap-D113-6578_bin88	28.4	5.2	0.9	74	Bacteria	Patescibacteria	Microgenomatia
Yap-D113-6578_bin89	27.3	6.8	0.7	162	Bacteria	Patescibacteria	Gracilibacteria
Yap-D113-6578_bin90	25.9	0.0	1.9	121	Bacteria	Bacteroidetes	Bacteroidia
Yap-D113-6578_bin91	25.2	1.0	1.7	45	Bacteria	unclassified	unclassified
Yap-D113-6578_bin92	25.0	3.5	0.9	522	Bacteria	Proteobacteria	unclassified
Yap-D113-6578_bin93	24.8	2.0	1.5	26	Bacteria	Patescibacteria	Paceibacteria
Yap-D113-6578_bin94	24.1	2.6	0.8	48	Bacteria	Patescibacteria	Microgenomatia
Yap-D113-6578_bin95	24.1	5.2	1.3	227	Bacteria	Omnitrophica	unclassified
Yap-D113-6578_bin96	24.1	4.5	0.9	105	Bacteria	Planctomycetes	Phycisphaerae
Yap-D113-6578_bin97	23.4	1.7	0.7	76	Bacteria	Patescibacteria	Paceibacteria
Yap-D113-6578_bin98	23.3	0.0	0.6	312	Bacteria	Planctomycetes	Phycisphaerae
Yap-D113-6578_bin99	23.3	6.9	0.6	217	Bacteria	Bacteroidetes	unclassified
Yap-D113-6578_bin100	23.2	1.7	0.7	53	Bacteria	Patescibacteria	Paceibacteria
Yap-D113-6578_bin101	22.4	0.0	1.7	396	Bacteria	Proteobacteria	Gammaproteobacteria
Yap-D113-6578_bin102	22.4	0.0	0.8	114	Bacteria	Zixibacteria	MSB-5A5
Yap-D113-6578_bin103	21.6	5.2	1.0	93	Bacteria	Dependentiae	Babeliae
Yap-D113-6578_bin104	21.4	0.0	0.8	139	Bacteria	Proteobacteria	Gammaproteobacteria
Yap-D113-6578_bin105	21.0	0.0	0.6	109	Bacteria	Chloroflexi	Dehalococcoidia
Yap-D113-6578_bin106	20.8	2.5	1.2	24	Bacteria	Patescibacteria	Paceibacteria
Yap-D113-6578_bin107	20.5	0.0	0.7	125	Bacteria	Chloroflexi	Anaerolineae
Yap-D113-6578_bin108	20.3	1.7	0.6	147	Bacteria	Omnitrophica	Omnitrophica
Yap-D113-6578_bin109	19.8	0.0	0.8	123	Bacteria	Proteobacteria	Gammaproteobacteria
Yap-D113-6578_bin110	19.0	1.7	1.0	103	Bacteria	Margulisbacteria	unclassified

Yap-D113-6578_bin111	19.0	5.2	0.6	134	Bacteria	Proteobacteria	Gammaproteobacteria
Yap-D113-6578_bin112	18.5	1.7	0.8	99	Bacteria	Chloroflexi	Dehalococcoidia
Yap-D113-6578_bin113	18.1	0.0	0.6	74	Bacteria	Proteobacteria	Gammaproteobacteria
Yap-D113-6578_bin114	17.9	6.7	0.5	68	Bacteria	Patescibacteria	Paceibacteria
Yap-D113-6578_bin115	17.6	0.0	0.9	515	Bacteria	Bacteroidetes	Bacteroidia
Yap-D113-6578_bin116	17.2	0.0	0.7	228	Bacteria	Omnitrophica	Omnitrophica
Yap-D113-6578_bin117	17.1	0.0	0.6	100	Bacteria	Nitrospinae	unclassified
Yap-D113-6578_bin118	17.1	6.9	0.7	265	Bacteria	SM23-31	SM23-31
Yap-D113-6578_bin119	17.0	0.0	0.5	463	Bacteria	unclassified	unclassified
Yap-D113-6578_bin120	17.0	0.0	0.7	115	Bacteria	Nanoarchaeota	unclassified
Yap-D113-6578_bin121	16.5	0.0	0.9	100	Bacteria	Proteobacteria	Alphaproteobacteria
Yap-D113-6578_bin122	15.9	0.0	0.4	66	Bacteria	Patescibacteria	Paceibacteria
Yap-D113-6578_bin123	15.8	0.2	0.4	654	Bacteria	unclassified	unclassified
Yap-D113-6578_bin124	15.7	0.2	0.8	407	Bacteria	Poribacteria	unclassified
Yap-D113-6578_bin125	15.7	1.7	1.1	229	Bacteria	Proteobacteria	Gammaproteobacteria
Yap-D113-6578_bin126	14.9	0.0	0.6	47	Bacteria	Patescibacteria	Paceibacteria
Yap-D113-6578_bin127	14.9	0.0	0.8	202	Bacteria	unclassified	unclassified
Yap-D113-6578_bin128	13.9	4.4	2.6	202	Bacteria	Dadabacteria	UBA1144
Yap-D113-6578_bin129	13.8	0.0	0.9	29	Bacteria	Dadabacteria	UBA1144
Yap-D113-6578_bin130	13.2	0.0	0.8	48	Bacteria	Fibrobacteres	unclassified
Yap-D113-6578_bin131	12.9	0.9	5.2	70	Bacteria	Bacteroidetes	Ignavibacteria
Yap-D113-6578_bin132	12.7	0.0	0.8	85	Bacteria	unclassified	unclassified
Yap-D113-6578_bin133	12.5	0.0	1.6	88	Bacteria	unclassified	unclassified
Yap-D113-6578_bin134	12.4	0.0	0.8	63	Bacteria	Bdellovibrionaeota	unclassified
Yap-D113-6578_bin135	12.1	0.0	0.8	75	Bacteria	unclassified	unclassified
Yap-D113-6578_bin136	12.0	0.0	0.4	116	Bacteria	unclassified	unclassified

Yap-D113-6578_bin137	11.9	0.2	0.8	76	Bacteria	Proteobacteria	Gammaproteobacteria
Yap-D113-6578_bin138	11.6	0.0	0.6	92	Bacteria	Chloroflexi	Dehalococcoidia
Yap-D113-6578_bin139	11.2	0.0	1.0	97	Bacteria	Proteobacteria	Gammaproteobacteria
Yap-D113-6578_bin140	10.5	3.5	0.6	77	Bacteria	Planctomycetes	Phycisphaerae
Yap-D113-6578_bin141	10.3	0.5	0.6	387	Bacteria	Proteobacteria	unclassified
Yap-D113-6578_bin142	9.9	0.0	0.4	352	Bacteria	unclassified	unclassified
Yap-D113-6578_bin143	9.9	0.0	0.9	64	Bacteria	unclassified	unclassified
Yap-D113-6578_bin144	9.5	2.6	0.3	109	Bacteria	Omnitrophica	koll11
Yap-D113-6578_bin145	8.7	0.0	2.3	44	Bacteria	Bacteroidetes	koll11
Yap-D113-6578_bin146	8.6	0.1	0.5	106	Bacteria	unclassified	unclassified
Yap-D113-6578_bin147	8.0	0.0	0.6	35	Bacteria	Patescibacteria	Microgenomatia
Yap-D113-6578_bin148	7.4	0.0	1.0	143	Bacteria	Nitrospirae	Nitrospiria
Yap-D113-6578_bin149	6.9	3.5	0.9	67	Bacteria	Chloroflexi	Dehalococcoidia
Yap-D113-6578_bin150	6.4	0.1	0.6	57	Bacteria	Elusimicrobia	unclassified
Yap-D113-6578_bin151	6.3	0.0	0.6	141	Bacteria	Actinobacteria	unclassified
Yap-D113-6578_bin152	5.6	1.8	1.3	114	Bacteria	Nitrospirae	Nitrospiria
Yap-D113-6578_bin153	4.2	0.0	0.7	33	Bacteria	Proteobacteria	Gammaproteobacteria
Yap-D113-6578_bin154	4.2	4.2	1.0	319	Bacteria	Proteobacteria	Gammaproteobacteria
Yap-D113-6578_bin155	3.9	0.1	0.6	151	Bacteria	Proteobacteria	Gammaproteobacteria
Yap-D113-6578_bin156	2.7	0.0	1.3	50	Bacteria	Fibrobacteres	unclassified
Yap-D113-6578_bin157	1.8	0.0	0.9	52	Bacteria	UBA10199	unclassified
Yap-D113-6578_bin158	0.0	0.0	0.9	37	Bacteria	unclassified	unclassified
Yap-D113-6578_bin159	22.9	3.3	0.5	179	unclassified	unclassified	unclassified
Yap-D113-6578_bin160	21.6	6.9	0.8	354	unclassified	unclassified	unclassified
Yap-D113-6578_bin161	6.3	0.0	1.3	63	unclassified	unclassified	unclassified
Yap-D113-6578_bin162	5.4	0.9	0.8	812	unclassified	unclassified	unclassified

Yap-CTD02-5000_bin1	90.3	22.8	9.6	212		Archaea	Thaumarchaeota	Nitrososphaeria
Yap-CTD02-5000_bin2	67.3	1.7	1.3	171		Archaea	Euryarchaeota	Thermoplasmata
Yap-CTD02-5000_bin3	61.2	1.9	0.9	77		Archaea	Thaumarchaeota	Nitrososphaeria
Yap-CTD02-5000_bin4	59.4	0.0	1.4	34		Archaea	Nanoarchaeota	Woesearchaeia
Yap-CTD02-5000_bin5	57.9	0.0	1.6	57		Archaea	Nanoarchaeota	Woesearchaeia
Yap-CTD02-5000_bin6	41.4	0.0	0.7	79		Archaea	UBA543	UBA543
Yap-CTD02-5000_bin7	30.7	1.0	2.9	166		Archaea	Thaumarchaeota	Nitrososphaeria
Yap-CTD02-5000_bin8	21.2	3.9	24.2	155		Archaea	Thaumarchaeota	Nitrososphaeria
Yap-CTD02-5000_bin9	18.1	0.1	1.0	105		Archaea	Euryarchaeota	Thermoplasmata
Yap-CTD02-5000_bin10	14.5	0.0	0.8	122		Archaea	Euryarchaeota	Thermoplasmata
Yap-CTD02-5000_bin11	11.1	0.0	1.9	12		Archaea	Nanoarchaeota	Woesearchaeia
Yap-CTD02-5000_bin12	9.4	0.0	0.8	72		Archaea	unclassified	unclassified
Yap-CTD02-5000_bin13	8.9	1.7	2.7	148		Archaea	Thaumarchaeota	Nitrososphaeria
Yap-CTD02-5000_bin14	7.1	1.0	3.7	65		Archaea	Thaumarchaeota	Nitrososphaeria
Yap-CTD02-5000_bin15	99.6	0.5	10.2	96		Bacteria	Bacteroidetes	Bacteroidia
Yap-CTD02-5000_bin16	98.4	1.3	18.3	143		Bacteria	Proteobacteria	Gammaproteobacteria
Yap-CTD02-5000_bin17	96.3	3.7	3.4	165		Bacteria	Bacteroidetes	Bacteroidia
Yap-CTD02-5000_bin18	92.3	3.9	5.5	194		Bacteria	Marinimicrobia	Marinisomatia
Yap-CTD02-5000_bin19	91.7	45.5	1.7	811		Bacteria	Proteobacteria	Gammaproteobacteria
Yap-CTD02-5000_bin20	91.5	0.2	43.3	71		Bacteria	Proteobacteria	Gammaproteobacteria
Yap-CTD02-5000_bin21	87.3	23.5	1.1	394		Bacteria	Planctomycetes	Phycisphaerae
Yap-CTD02-5000_bin22	86.9	4.9	2.5	217		Bacteria	Planctomycetes	Phycisphaerae
Yap-CTD02-5000_bin23	81.9	0.8	1.3	212		Bacteria	Firmicutes	Bacilli
Yap-CTD02-5000_bin24	80.0	8.8	4.8	875		Bacteria	Proteobacteria	Gammaproteobacteria
Yap-CTD02-5000_bin25	75.8	2.2	3.6	351		Bacteria	Marinimicrobia	Marinisomatia
Yap-CTD02-5000_bin26	74.4	4.1	1.1	368		Bacteria	Proteobacteria	Gammaproteobacteria

Yap-CTD02-5000_bin27	72.0	12.6	2.2	221	Bacteria	Marinimicrobia	Marinisomatia
Yap-CTD02-5000_bin28	70.9	6.4	0.9	432	Bacteria	Nitrospirae	Nitrospiria
Yap-CTD02-5000_bin29	69.6	1.1	7.0	358	Bacteria	SAR324	SAR324
Yap-CTD02-5000_bin30	69.1	4.7	4.4	334	Bacteria	Firmicutes	Bacilli
Yap-CTD02-5000_bin31	68.8	3.6	2.2	156	Bacteria	Proteobacteria	Gammaproteobacteria
Yap-CTD02-5000_bin32	68.2	5.5	0.9	351	Bacteria	Dadabacteria	UBA1144
Yap-CTD02-5000_bin33	68.1	1.2	3.5	235	Bacteria	Marinimicrobia	Marinisomatia
Yap-CTD02-5000_bin34	65.4	3.4	1.0	514	Bacteria	Marinimicrobia	Marinisomatia
Yap-CTD02-5000_bin35	64.6	1.5	1.7	82	Bacteria	Patescibacteria	Paceibacteria
Yap-CTD02-5000_bin36	64.6	17.6	1.5	496	Bacteria	Marinimicrobia	Marinisomatia
Yap-CTD02-5000_bin37	63.2	7.0	5.6	206	Bacteria	Proteobacteria	Gammaproteobacteria
Yap-CTD02-5000_bin38	54.8	5.2	0.7	302	Bacteria	Planctomycetes	Phycisphaerae
Yap-CTD02-5000_bin39	48.7	11.1	0.8	75	Bacteria	Patescibacteria	Paceibacteria
Yap-CTD02-5000_bin40	47.5	0.0	0.9	893	Bacteria	Firmicutes	Bacilli
Yap-CTD02-5000_bin41	44.8	1.7	0.9	493	Bacteria	Proteobacteria	Gammaproteobacteria
Yap-CTD02-5000_bin42	44.3	3.3	1.1	155	Bacteria	Marinimicrobia	Marinisomatia
Yap-CTD02-5000_bin43	43.8	0.7	1.1	318	Bacteria	Firmicutes	Bacilli
Yap-CTD02-5000_bin44	43.8	8.4	6.3	182	Bacteria	Proteobacteria	Alphaproteobacteria
Yap-CTD02-5000_bin45	43.0	8.4	1.0	508	Bacteria	Chloroflexi	Dehalococcoidia
Yap-CTD02-5000_bin46	40.7	4.3	0.9	350	Bacteria	SAR324	SAR324
Yap-CTD02-5000_bin47	39.7	0.0	1.1	387	Bacteria	Proteobacteria	Gammaproteobacteria
Yap-CTD02-5000_bin48	39.6	5.9	1.9	152	Bacteria	Proteobacteria	Gammaproteobacteria
Yap-CTD02-5000_bin49	36.2	0.0	0.7	637	Bacteria	Proteobacteria	Gammaproteobacteria
Yap-CTD02-5000_bin50	35.3	5.7	0.9	179	Bacteria	Proteobacteria	Alphaproteobacteria
Yap-CTD02-5000_bin51	35.0	2.0	0.8	274	Bacteria	Chloroflexi	Dehalococcoidia
Yap-CTD02-5000_bin52	34.5	4.4	11.7	295	Bacteria	Proteobacteria	Alphaproteobacteria

Yap-CTD02-5000_bin53	34.3	4.3	0.7	128	Bacteria	Dadabacteria	UBA1144
Yap-CTD02-5000_bin54	22.7	0.0	0.8	405	Bacteria	Proteobacteria	Gammaproteobacteria
Yap-CTD02-5000_bin55	22.3	0.0	0.6	164	Bacteria	Planctomycetes	Phycisphaerae
Yap-CTD02-5000_bin56	19.1	0.4	0.7	364	Bacteria	Proteobacteria	Gammaproteobacteria
Yap-CTD02-5000_bin57	17.9	0.1	51.0	126	Bacteria	Proteobacteria	Gammaproteobacteria
Yap-CTD02-5000_bin58	17.2	1.7	0.7	312	Bacteria	Bacteroidetes	Bacteroidia
Yap-CTD02-5000_bin59	16.8	0.1	36.8	90	Bacteria	Marinimicrobia	Marinisomatia
Yap-CTD02-5000_bin60	16.2	1.2	0.8	170	Bacteria	Proteobacteria	Gammaproteobacteria
Yap-CTD02-5000_bin61	16.0	0.0	0.7	251	Bacteria	Proteobacteria	Gammaproteobacteria
Yap-CTD02-5000_bin62	15.8	0.0	1.1	85	Bacteria	Proteobacteria	Gammaproteobacteria
Yap-CTD02-5000_bin63	15.7	0.3	0.7	368	Bacteria	Chloroflexi	Dehalococcoidia
Yap-CTD02-5000_bin64	15.5	0.0	1.7	218	Bacteria	Proteobacteria	Gammaproteobacteria
Yap-CTD02-5000_bin65	12.8	3.5	1.0	113	Bacteria	Planctomycetes	Phycisphaerae
Yap-CTD02-5000_bin66	12.7	0.0	0.6	47	Bacteria	Patescibacteria	Paceibacteria
Yap-CTD02-5000_bin67	10.8	0.0	1.8	100	Bacteria	Bacteroidetes	Bacteroidia
Yap-CTD02-5000_bin68	10.3	0.0	0.8	52	Bacteria	Bacteroidetes	Bacteroidia
Yap-CTD02-5000_bin69	10.3	1.7	1.8	40	Bacteria	Bacteroidetes	Bacteroidia
Yap-CTD02-5000_bin70	7.8	0.0	2.6	58	Bacteria	Proteobacteria	Alphaproteobacteria
Yap-CTD02-5000_bin71	5.5	0.0	0.9	101	Bacteria	Marinimicrobia	Gammaproteobacteria
Yap-CTD02-5000_bin72	5.3	0.0	1.1	327	Bacteria	Verrucomicrobia	Verrucomicrobiae
Yap-CTD02-5000_bin73	4.2	0.0	0.7	90	Bacteria	Marinimicrobia	Marinisomatia
Yap-CTD02-5000_bin74	3.7	0.0	1.2	1817	Bacteria	Proteobacteria	Gammaproteobacteria
Yap-CTD02-5000_bin75	2.1	0.0	2.0	299	Bacteria	Proteobacteria	Gammaproteobacteria
Yap-CTD02-5000_bin76	1.3	0.0	0.7	18	Bacteria	Chloroflexi	Dehalococcoidia
Yap-CTD02-5000_bin77	9.8	0.0	1.9	357	unclassified	unclassified	unclassified
Yap-CTD02-5000_bin78	8.9	0.2	0.6	22829	unclassified	unclassified	unclassified

Yap-CTD02-5000_bin79	2.9	0.0	2.7	69	unclassified	unclassified	unclassified
Yap-CTD03-5700_bin1	97.8	26.1	3.5	187	Archaea	Thaumarchaeota	Nitrososphaeria
Yap-CTD03-5700_bin2	66.4	0.0	1.1	244	Archaea	Euryarchaeota	Thermoplasmata
Yap-CTD03-5700_bin3	30.4	0.0	0.6	181	Archaea	Euryarchaeota	Thermoplasmata
Yap-CTD03-5700_bin4	21.4	0.0	9.5	71	Archaea	Thaumarchaeota	Nitrososphaeria
Yap-CTD03-5700_bin5	13.5	0.0	0.6	167	Archaea	Euryarchaeota	Thermoplasmata
Yap-CTD03-5700_bin6	12.6	0.9	1.1	47	Archaea	Euryarchaeota	Thermoplasmata
Yap-CTD03-5700_bin7	99.3	3.0	3.1	26	Bacteria	Bacteroidetes	Bacteroidia
Yap-CTD03-5700_bin8	98.8	0.7	14.5	125	Bacteria	Proteobacteria	Gammaproteobacteria
Yap-CTD03-5700_bin9	98.5	6.8	16.0	51	Bacteria	Proteobacteria	Gammaproteobacteria
Yap-CTD03-5700_bin10	98.5	1.2	1.6	70	Bacteria	Proteobacteria	Gammaproteobacteria
Yap-CTD03-5700_bin11	98.4	0.1	1.5	67	Bacteria	Proteobacteria	Gammaproteobacteria
Yap-CTD03-5700_bin12	98.2	0.4	2.4	53	Bacteria	Proteobacteria	Alphaproteobacteria
Yap-CTD03-5700_bin13	97.2	1.9	2.2	370	Bacteria	Proteobacteria	Gammaproteobacteria
Yap-CTD03-5700_bin14	97.0	3.0	3.8	66	Bacteria	Proteobacteria	Gammaproteobacteria
Yap-CTD03-5700_bin15	96.8	0.2	2.2	71	Bacteria	Proteobacteria	Alphaproteobacteria
Yap-CTD03-5700_bin16	96.6	0.4	1.8	44	Bacteria	Bacteroidetes	Bacteroidia
Yap-CTD03-5700_bin17	95.6	0.2	16.8	53	Bacteria	Proteobacteria	Gammaproteobacteria
Yap-CTD03-5700_bin18	92.6	3.6	2.3	451	Bacteria	Proteobacteria	Gammaproteobacteria
Yap-CTD03-5700_bin19	91.8	6.6	1.8	238	Bacteria	Marinimicrobia	Marinisomatia
Yap-CTD03-5700_bin20	89.3	2.0	2.3	362	Bacteria	SAR324	SAR324
Yap-CTD03-5700_bin21	87.2	18.4	4.4	663	Bacteria	Proteobacteria	Gammaproteobacteria
Yap-CTD03-5700_bin22	83.0	3.4	1.4	135	Bacteria	Proteobacteria	Gammaproteobacteria
Yap-CTD03-5700_bin23	81.3	2.3	2.0	251	Bacteria	Planctomycetes	Phycisphaerae
Yap-CTD03-5700_bin24	79.4	4.3	0.9	698	Bacteria	Nitrospirae	Nitrospiria
Yap-CTD03-5700_bin25	78.6	19.6	1.8	340	Bacteria	Proteobacteria	Gammaproteobacteria

Yap-CTD03-5700_bin26	75.8	0.8	0.8	302	Bacteria	Proteobacteria	Gammaproteobacteria
Yap-CTD03-5700_bin27	73.1	2.2	1.2	385	Bacteria	Marinimicrobia	Marinisomatia
Yap-CTD03-5700_bin28	71.0	0.0	1.6	259	Bacteria	Marinimicrobia	Marinisomatia
Yap-CTD03-5700_bin29	65.4	4.0	0.9	216	Bacteria	Planctomycetes	Phycisphaerae
Yap-CTD03-5700_bin30	63.2	2.6	5.2	285	Bacteria	Firmicutes	Bacilli
Yap-CTD03-5700_bin31	61.8	0.8	1.0	448	Bacteria	Proteobacteria	Gammaproteobacteria
Yap-CTD03-5700_bin32	58.7	5.2	2.1	314	Bacteria	Proteobacteria	Gammaproteobacteria
Yap-CTD03-5700_bin33	58.5	1.3	0.8	85	Bacteria	Patescibacteria	Paceibacteria
Yap-CTD03-5700_bin34	58.4	6.6	1.8	177	Bacteria	Proteobacteria	Alphaproteobacteria
Yap-CTD03-5700_bin35	57.6	4.5	14.7	447	Bacteria	Marinimicrobia	Marinisomatia
Yap-CTD03-5700_bin36	57.5	15.8	0.7	663	Bacteria	Bacteroidetes	Bacteroidia
Yap-CTD03-5700_bin37	57.5	3.1	2.5	201	Bacteria	Proteobacteria	Gammaproteobacteria
Yap-CTD03-5700_bin38	54.4	10.4	0.9	197	Bacteria	Bacteroidetes	Bacteroidia
Yap-CTD03-5700_bin39	53.5	6.1	0.7	377	Bacteria	Marinimicrobia	Marinisomatia
Yap-CTD03-5700_bin40	51.9	2.3	0.7	336	Bacteria	Bacteroidetes	Bacteroidia
Yap-CTD03-5700_bin41	50.9	12.5	7.4	708	Bacteria	Proteobacteria	Gammaproteobacteria
Yap-CTD03-5700_bin42	48.1	2.7	0.7	178	Bacteria	Planctomycetes	Phycisphaerae
Yap-CTD03-5700_bin43	47.6	8.6	9.0	13	Bacteria	Proteobacteria	Gammaproteobacteria
Yap-CTD03-5700_bin44	46.9	1.4	0.8	273	Bacteria	Proteobacteria	Gammaproteobacteria
Yap-CTD03-5700_bin45	44.0	1.7	0.9	295	Bacteria	Firmicutes	Bacilli
Yap-CTD03-5700_bin46	40.6	6.9	1.0	536	Bacteria	Proteobacteria	Gammaproteobacteria
Yap-CTD03-5700_bin47	38.8	1.7	0.9	365	Bacteria	Proteobacteria	Alphaproteobacteria
Yap-CTD03-5700_bin48	38.6	1.3	0.6	455	Bacteria	Firmicutes	Bacilli
Yap-CTD03-5700_bin49	36.2	0.0	0.6	600	Bacteria	Bacteroidetes	Bacteroidia
Yap-CTD03-5700_bin50	35.4	0.2	3.8	238	Bacteria	Marinimicrobia	Marinisomatia
Yap-CTD03-5700_bin51	34.8	1.7	0.9	428	Bacteria	Proteobacteria	Gammaproteobacteria

Yap-CTD03-5700_bin52	34.3	1.1	0.8	138	Bacteria	Planctomycetes	Phycisphaerae
Yap-CTD03-5700_bin53	34.1	1.7	9.3	67	Bacteria	Proteobacteria	Gammaproteobacteria
Yap-CTD03-5700_bin54	32.8	0.0	0.9	479	Bacteria	Proteobacteria	Gammaproteobacteria
Yap-CTD03-5700_bin55	31.5	1.7	0.7	156	Bacteria	Marinimicrobia	Marinisomatia
Yap-CTD03-5700_bin56	31.3	0.0	0.9	236	Bacteria	Proteobacteria	Gammaproteobacteria
Yap-CTD03-5700_bin57	30.1	3.5	0.5	554	Bacteria	Chloroflexi	Dehalococcoidia
Yap-CTD03-5700_bin58	29.3	0.0	0.8	140	Bacteria	Bacteroidetes	Bacteroidia
Yap-CTD03-5700_bin59	29.2	0.0	0.7	299	Bacteria	Proteobacteria	Alphaproteobacteria
Yap-CTD03-5700_bin60	27.7	0.0	0.7	480	Bacteria	Bacteroidetes	Bacteroidia
Yap-CTD03-5700_bin61	25.9	5.2	1.1	77	Bacteria	Planctomycetes	Phycisphaerae
Yap-CTD03-5700_bin62	23.2	0.5	0.7	249	Bacteria	Proteobacteria	Gammaproteobacteria
Yap-CTD03-5700_bin63	23.2	1.7	0.6	104	Bacteria	Acidobacteria	unclassified
Yap-CTD03-5700_bin64	20.2	0.0	7.0	250	Bacteria	Proteobacteria	Gammaproteobacteria
Yap-CTD03-5700_bin65	19.6	0.0	0.6	263	Bacteria	Proteobacteria	Gammaproteobacteria
Yap-CTD03-5700_bin66	17.9	0.0	4.0	79	Bacteria	Proteobacteria	Alphaproteobacteria
Yap-CTD03-5700_bin67	15.6	0.0	1.1	107	Bacteria	Proteobacteria	Gammaproteobacteria
Yap-CTD03-5700_bin68	15.6	0.7	0.6	139	Bacteria	Verrucomicrobia	Verrucomicrobiae
Yap-CTD03-5700_bin69	13.8	0.0	1.4	109	Bacteria	Proteobacteria	Gammaproteobacteria
Yap-CTD03-5700_bin70	12.1	0.0	7.1	149	Bacteria	Firmicutes	Bacilli
Yap-CTD03-5700_bin71	11.8	0.9	1.7	102	Bacteria	Proteobacteria	Gammaproteobacteria
Yap-CTD03-5700_bin72	11.7	0.0	0.7	177	Bacteria	Planctomycetes	Phycisphaerae
Yap-CTD03-5700_bin73	11.6	0.0	0.6	62	Bacteria	Proteobacteria	Alphaproteobacteria
Yap-CTD03-5700_bin74	10.0	0.0	0.5	305	Bacteria	Chloroflexi	Dehalococcoidia
Yap-CTD03-5700_bin75	9.4	0.7	0.4	58	Bacteria	Planctomycetes	Phycisphaerae
Yap-CTD03-5700_bin76	8.8	0.0	1.4	104	Bacteria	Marinimicrobia	Marinisomatia
Yap-CTD02-6000_bin1	47.3	0.0	0.7	51	Archaea	Nanoarchaeota	Woesearchaeia

Yap-CTD02-6000_bin2	44.6	2.4	5.5	109	Archaea	Thaumarchaeota	Nitrososphaeria
Yap-CTD02-6000_bin3	35.3	0.0	0.6	62	Archaea	Nanoarchaeota	Woesearchaeia
Yap-CTD02-6000_bin4	32.9	0.0	0.6	236	Archaea	Euryarchaeota	Thermoplasmata
Yap-CTD02-6000_bin5	29.8	1.2	5.8	68	Archaea	Thaumarchaeota	Nitrososphaeria
Yap-CTD02-6000_bin6	27.3	1.0	8.2	66	Archaea	Thaumarchaeota	Nitrososphaeria
Yap-CTD02-6000_bin7	17.3	1.9	11.6	73	Archaea	Thaumarchaeota	Nitrososphaeria
Yap-CTD02-6000_bin8	5.3	0.0	3.7	14	Archaea	Thaumarchaeota	Nitrososphaeria
Yap-CTD02-6000_bin9	100.0	0.0	9.6	34	Bacteria	Proteobacteria	Gammaproteobacteria
Yap-CTD02-6000_bin10	100.0	0.2	11.5	54	Bacteria	Proteobacteria	Gammaproteobacteria
Yap-CTD02-6000_bin11	99.9	0.9	2.3	34	Bacteria	Proteobacteria	Gammaproteobacteria
Yap-CTD02-6000_bin12	98.7	1.6	10.4	88	Bacteria	Proteobacteria	Gammaproteobacteria
Yap-CTD02-6000_bin13	97.3	11.8	4.0	629	Bacteria	Proteobacteria	Gammaproteobacteria
Yap-CTD02-6000_bin14	97.0	4.3	46.5	200	Bacteria	Proteobacteria	Gammaproteobacteria
Yap-CTD02-6000_bin15	95.3	3.5	2.4	108	Bacteria	Bacteroidetes	Bacteroidia
Yap-CTD02-6000_bin16	94.7	1.7	1.8	181	Bacteria	Proteobacteria	Gammaproteobacteria
Yap-CTD02-6000_bin17	92.9	5.4	3.1	448	Bacteria	Proteobacteria	Gammaproteobacteria
Yap-CTD02-6000_bin18	92.0	21.9	3.8	536	Bacteria	Proteobacteria	Gammaproteobacteria
Yap-CTD02-6000_bin19	89.8	2.9	1.5	225	Bacteria	Planctomycetes	Phycisphaerae
Yap-CTD02-6000_bin20	86.8	1.1	1.2	281	Bacteria	SAR324	SAR324
Yap-CTD02-6000_bin21	83.8	1.7	1.6	152	Bacteria	Marinimicrobia	Marinisomatia
Yap-CTD02-6000_bin22	81.2	2.0	2.0	71	Bacteria	Proteobacteria	Gammaproteobacteria
Yap-CTD02-6000_bin23	81.1	8.5	2.9	1053	Bacteria	Proteobacteria	Gammaproteobacteria
Yap-CTD02-6000_bin24	78.6	2.3	1.3	243	Bacteria	Marinimicrobia	Marinisomatia
Yap-CTD02-6000_bin25	75.8	10.1	0.7	619	Bacteria	Dadabacteria	UBA1144
Yap-CTD02-6000_bin26	70.6	3.9	0.8	479	Bacteria	Marinimicrobia	Marinisomatia
Yap-CTD02-6000_bin27	69.0	0.0	0.8	669	Bacteria	Proteobacteria	Gammaproteobacteria

Yap-CTD02-6000_bin28	67.0	0.0	0.8	353	Bacteria	Bacteroidetes	Bacteroidia
Yap-CTD02-6000_bin29	66.0	8.1	10.7	357	Bacteria	Marinimicrobia	Marinisomatia
Yap-CTD02-6000_bin30	64.8	27.9	0.7	747	Bacteria	Proteobacteria	unclassified
Yap-CTD02-6000_bin31	63.7	2.5	1.1	96	Bacteria	Patescibacteria	Paceibacteria
Yap-CTD02-6000_bin32	63.5	1.7	3.9	256	Bacteria	Proteobacteria	Gammaproteobacteria
Yap-CTD02-6000_bin33	61.9	0.4	1.7	359	Bacteria	Proteobacteria	Gammaproteobacteria
Yap-CTD02-6000_bin34	61.4	4.3	1.1	274	Bacteria	Proteobacteria	Gammaproteobacteria
Yap-CTD02-6000_bin35	59.6	2.0	4.1	311	Bacteria	Firmicutes	Bacilli
Yap-CTD02-6000_bin36	58.2	3.5	0.7	397	Bacteria	Bacteroidetes	Bacteroidia
Yap-CTD02-6000_bin37	47.2	4.4	0.6	419	Bacteria	Planctomycetes	Phycisphaerae
Yap-CTD02-6000_bin38	47.0	1.7	166.3	604	Bacteria	Proteobacteria	Gammaproteobacteria
Yap-CTD02-6000_bin39	44.8	2.1	0.7	393	Bacteria	Proteobacteria	Alphaproteobacteria
Yap-CTD02-6000_bin40	44.0	3.6	3.8	189	Bacteria	Proteobacteria	Alphaproteobacteria
Yap-CTD02-6000_bin41	42.6	2.9	1.1	193	Bacteria	Proteobacteria	Gammaproteobacteria
Yap-CTD02-6000_bin42	37.1	4.3	0.8	223	Bacteria	Acidobacteria	unclassified
Yap-CTD02-6000_bin43	35.4	4.2	1.1	215	Bacteria	Bacteroidetes	Bacteroidia
Yap-CTD02-6000_bin44	33.6	0.5	0.7	231	Bacteria	Proteobacteria	Gammaproteobacteria
Yap-CTD02-6000_bin45	31.7	2.2	0.6	173	Bacteria	Marinimicrobia	Marinisomatia
Yap-CTD02-6000_bin46	31.0	6.9	2.1	228	Bacteria	Firmicutes	Bacilli
Yap-CTD02-6000_bin47	30.5	7.7	4.3	378	Bacteria	Marinimicrobia	Marinisomatia
Yap-CTD02-6000_bin48	29.5	0.0	0.5	84	Bacteria	Proteobacteria	Gammaproteobacteria
Yap-CTD02-6000_bin49	28.1	0.0	0.5	160	Bacteria	Planctomycetes	Phycisphaerae
Yap-CTD02-6000_bin50	26.7	0.0	0.5	211	Bacteria	Proteobacteria	Gammaproteobacteria
Yap-CTD02-6000_bin51	25.9	1.9	0.5	229	Bacteria	Proteobacteria	Alphaproteobacteria
Yap-CTD02-6000_bin52	25.5	5.0	0.7	318	Bacteria	Proteobacteria	Gammaproteobacteria
Yap-CTD02-6000_bin53	25.0	0.0	0.5	213	Bacteria	Bacteroidetes	Bacteroidia

Yap-CTD02-6000_bin54	23.0	6.0	2.1	70	Bacteria	Proteobacteria	Alphaproteobacteria
Yap-CTD02-6000_bin55	22.9	0.0	0.6	392	Bacteria	Nitrospirae	Nitrospiria
Yap-CTD02-6000_bin56	21.8	0.0	1.7	251	Bacteria	Proteobacteria	Gammaproteobacteria
Yap-CTD02-6000_bin57	20.7	0.0	0.5	374	Bacteria	Bacteroidetes	Bacteroidia
Yap-CTD02-6000_bin58	20.7	10.5	2.8	26	Bacteria	Proteobacteria	Gammaproteobacteria
Yap-CTD02-6000_bin59	17.4	0.0	5.4	162	Bacteria	Proteobacteria	Alphaproteobacteria
Yap-CTD02-6000_bin60	17.2	0.0	1.5	38	Bacteria	Proteobacteria	Gammaproteobacteria
Yap-CTD02-6000_bin61	16.0	0.0	1.7	127	Bacteria	Proteobacteria	Gammaproteobacteria
Yap-CTD02-6000_bin62	15.3	0.9	0.7	332	Bacteria	Proteobacteria	Alphaproteobacteria
Yap-CTD02-6000_bin63	15.2	0.0	0.9	208	Bacteria	Bacteroidetes	Bacteroidia
Yap-CTD02-6000_bin64	14.7	0.0	0.4	311	Bacteria	Firmicutes	Bacilli
Yap-CTD02-6000_bin65	14.3	1.9	2.3	156	Bacteria	Proteobacteria	Alphaproteobacteria
Yap-CTD02-6000_bin66	12.5	5.2	2.4	79	Bacteria	Marinimicrobia	Marinisomatia
Yap-CTD02-6000_bin67	11.2	0.0	0.5	358	Bacteria	Thaumarchaeota	Nitrososphaeria
Yap-CTD02-6000_bin68	6.5	0.0	0.5	188	Bacteria	Proteobacteria	Alphaproteobacteria
Yap-CTD02-6000_bin69	4.2	0.0	9.4	49	Bacteria	Marinimicrobia	Marinisomatia
Yap-CTD02-6000_bin70	4.2	0.0	1.6	95	Bacteria	Bacteroidetes	Bacteroidia
Yap-CTD02-6000_bin71	1.4	0.0	0.5	71	Bacteria	Proteobacteria	Alphaproteobacteria
Yap-CTD02-6000_bin72	1.0	0.0	0.7	552	Bacteria	Bacteroidetes	Bacteroidia
